# The Glucocorticoid Receptor Polymorphism Landscape in Patients With Diamond Blackfan Anemia Reveals an Association Between Two Clinically Relevant Single Nucleotide Polymorphisms and Time to Diagnosis

**DOI:** 10.3389/fphys.2021.745032

**Published:** 2021-10-13

**Authors:** Annalisa Lonetti, Valentina Indio, Irma Dianzani, Ugo Ramenghi, Lydie Da Costa, Dagmar Pospíšilová, Anna Rita Migliaccio

**Affiliations:** ^1^Department of Biomedical and Neuromotor Sciences, University of Bologna, Bologna, Italy; ^2^Giorgio Prodi Cancer Research Center, University of Bologna, Bologna, Italy; ^3^Department of Health Sciences, Università del Piemonte Orientale, Novara, Italy; ^4^Department of Public Health and Pediatric Sciences, University of Turin, Turin, Italy; ^5^Service d’Hématologie Biologique, Hôpital Robert Debré, University of Paris, Paris, France; ^6^Department of Pediatrics, Faculty Hospital of Palacky University, Olomouc, Czechia; ^7^Faculty of Medicine and Surgery, University Campus Bio-Medico, Rome, Italy

**Keywords:** Diamond Blackfan anemia, glucocorticoid receptor, single nucleotide polymorphisms, glucocorticoid response, time to diagnosis

## Abstract

NR3C1, the gene encoding the glucocorticoid receptor, is polymorphic presenting numerous single nucleotide polymorphisms (SNPs) some of which are emerging as leading cause in the variability of manifestation and/or response to glucocorticoids in human diseases. Since 60–80% of patients with Diamond Blackfan anemia (DBA), an inherited pure red cell aplasia induced by mutations in ribosomal protein genes became transfusion independent upon treatment with glucocorticoids, we investigated whether clinically relevant NR3C1 SNPs are associated with disease manifestation in DBA. The eight SNPs rs10482605, rs10482616, rs7701443, rs6189/rs6190, rs860457, rs6198, rs6196, and rs33388/rs33389 were investigated in a cohort of 91 European DBA patients. Results were compared with those observed in healthy volunteers (*n*=37) or present in public genome databases of Italian and European populations. Although, cases vs. control analyses suggest that the frequency of some of the minor alleles is significantly altered in DBA patients with respect to healthy controls or to the Italian or other European registries, lack of consistency among the associations across different sets suggests that overall the frequency of these SNPs in DBA is not different from that of the general population. Demographic data (47 females and 31 males) and driver mutations (44 S and 29 L genes and eight no-known mutation) are known for 81 patients while glucocorticoid response is known, respectively, for 81 (36 responsive and 45 non-responsive) and age of disease onsets for 79 (55 before and 24 after 4months of age) patients. Neither gender nor leading mutations were associated with the minor alleles or with disease manifestation. In addition, none of the SNPs met the threshold in the response vs. non-responsive groups. However, two SNPs (rs6196 and rs860457) were enriched in patients manifesting the disease before 4months of age. Although the exact biomechanistical consequences of these SNPs are unknown, the fact that their configuration is consistent with that of regulatory regions suggests that they regulate changes in glucocorticoid response during ontogeny. This hypothesis was supported by phosphoproteomic profiling of erythroid cells expanded *ex vivo* indicating that glucocorticoids activate a ribosomal signature in cells from cord blood but not in those from adult blood, possibly providing a compensatory mechanism to the driving mutations observed in DBA before birth.

## Introduction

Diamond Blackfan anemia (DBA) is an inherited bone marrow failure syndrome (Vlachos et al., 2008). At birth, DBA patients are usually not anemic and, for reason still unknown, the disease manifests itself at a wide range of age with some patients developing anemia few weeks after birth while others becoming anemic only in adulthood (Balaban et al., 1985; Vlachos et al., 2008). Disease manifestation includes normochromic macrocytic anemia, reticulocytopenia, limited cytopenia of other lineages, and a visible paucity of erythroid precursor cells in the bone marrow. A portion of the patients also shows congenital malformations and low stature (Vlachos et al., 2008). DBA is due to mutations in at least 23 different DBA/DBA-like genes. Most of the mutant genes encode ribosomal proteins, the most frequent being RPS19, RPL5, RPL11, RPS26, and RPS10 (Ulirsch et al., 2018). Each patient carries a single mutation and the disease is autosomal dominant with variable expressivity and incomplete penetrance (Vlachos et al., 2008). The mutations are loss of function and cause ribosomal stress in erythroid progenitors, whose consequences are decrease of general and specific protein synthesis (including genes essential for erythroid differentiation; Dutt et al., 2011; Horos et al., 2012; Moniz et al., 2012; Ludwig et al., 2014; Aspesi et al., 2017) and activation of apoptosis through p53-dependent and p53-independent mechanisms (Fumagalli et al., 2009; Aspesi et al., 2014; Ellis, 2014).

Recent retrospective analysis of a large population of DBA patients recruited in the Italian Registry indicates that patients carrying mutations in large ribosomal subunit protein L genes have a significant more severe phenotype, with the incidence of malformations, higher erythrocyte adenosine deaminase activity, and more severe outcomes, than patients with mutations in small ribosomal subunit protein S genes (Quarello et al., 2020).

Currently, hematopoietic stem cell transplantation is the sole curative option (Vlachos et al., 2008). However, although this procedure is highly effective and with limited risk especially if performed before 10years of age, it is generally restricted to the few patients with fully matched donor. Therefore, the current standard of care for DBA includes corticosteroids and/or chronic transfusions. Since treatment with corticosteroids is not advised for young infants (before 12months of age), DBA patients are always treated with transfusion if the disease onset is during the first year of life. Glucocorticoid therapy is started after the first year of age and improves anemia in 60–80% of the patients (Vlachos et al., 2008). However, this therapy has osteoporosis and growth impairment as its most important side effects and several patients became unresponsive to therapy with time. Glucocorticoid-unresponsive patients are treated with regular red blood cell transfusion and iron chelators, to reduce the toxicity of iron overload, all their life (Vlachos et al., 2008). To improve the treatment options for DBA patients, there is active research to identify novel less toxic and possibly more potent synthetic glucocorticoids and on the molecular mechanisms that leads to glucocorticoid resistance in this disease (Da Costa et al., 2020).

Glucocorticoids exert their biological functions by binding to a specific receptor, the glucocorticoid receptors (GR), present in the cytoplasm which, once activated by binding to its ligand, translocates to the nucleus to exert its transcriptional activity (Zhou and Cidlowski, 2005; Chen et al., 2008). The human glucocorticoid receptor is encoded by the *NR3C1* gene located on chromosome 5q31-32 that is expressed at high levels in cells of the liver, muscle, and vasculature (Francke and Foellmer, 1989; Encio and Detera-Wadleigh, 1991). The most prominent roles of this protein are the regulation of body metabolism and cardiovascular function (Zhou and Cidlowski, 2005). *NR3C1* genetic variants may alter the glucocorticoid response of these physiological systems by altering GR protein functions (Kraemer and Ratamess, 2005; Walker, 2007; Spijker and Van Rossum, 2009; Ash et al., 2016). In adult muscle cells and in erythroid progenitor cells, GR activation regulates mRNA translation by inducing REDD1 that, by suppressing mTOR, reduces 4E-BP1(S65) and S6KA1(S235/236) inhibiting initiation of mRNA translation and protein biosynthesis (Wang et al., 2006 and further discussed later in this article). It is then counterintuitive that GR agonists are effective in 60–80% of patients with DBA, an inherited form of pure RBC aplasia induced by mutations that impair protein biosynthesis by reducing the synthesis of new ribosomes.

The polymorphism of *NR3C1* includes the presence of large numbers of single nucleotide polymorphisms (SNPs), which have been associated with disease manifestation and/or glucocorticoid response in several human diseases (Zhou and Cidlowski, 2005; Varricchio and Migliaccio, 2014). The SNPs more frequently associated with human diseases are: rs10482605, that decreases the expression of the major binding isoform of GR (GRα) and predicts GC resistance in major depression (Spijker and Van Rossum, 2009; Anacker et al., 2013); rs10482616, rs7701443, rs6189/rs6190, rs860457, and rs6198, the biological consequences of which are unknown and that predict GC resistance in Crohn’s Disease (Krupoves et al., 2011); rs6196, that enhances the expression of the dominant negative GR isoform GRβ and predisposes to autoimmune diseases (Derijk et al., 2001; Rai et al., 2006; Piotrowski et al., 2007) and myeloproliferative neoplasms (Poletto et al., 2012; Varricchio and Migliaccio, 2014) while reducing the risk of diabetes in patients with Cushing’s Syndrome (Trementino et al., 2012), and rs33388/rs33389, that increases expression of the GC binding isoform GRγ, which binds GC 10-times less efficiently than GRα and has not been associated with human diseases as yet (Kraemer and Ratamess, 2005; Walker, 2007; Spijker and Van Rossum, 2009; Ash et al., 2016).

In spite of the great relevance of GR for the treatment of DBA, systematic studies on the frequency of GR polymorphisms and on their correlation with disease manifestation in this patient population have not been done as yet. In a previous study using patients of the Italian registry, we found that the rs6198 SNP is present at a frequency greater than normal in DBA (Varricchio et al., 2011). Incomplete clinical information on these patients did not allow us to perform correlative analyses with disease manifestation.

The aim of the present study was to confirm this previous finding using a larger cohort of patients and to identify whether the additional clinically relevant SNPs described above would also be enriched in this population. We aimed also to analyze whether any of these SNPs was correlated with disease manifestation in terms of response to treatment and time to disease manifestation in DBA.

## Materials and Methods

### Human Subjects and DNA Isolation

Peripheral blood was collected from 91 DBA patients and 37 healthy donors by clinical centers in Italy, France, and the Czech Republic in accordance with guidelines established by the local ethics committees for human subject studies as recommended by the Declaration of Helsinki (Supplementary Table S1). The DBA patients included in the study were the subject of previous publications. DBA patients from the Italian registry were published in Varricchio et al. (2011) that compared the frequency of the rs6198 SNPs in patients and healthy controls. The additional DBA patients were published in Horos et al. (2012) to compare the response to glucocorticoids between patients and healthy controls. DNA was isolated from mononuclear blood cells isolated by Ficoll-Hypaque centrifugation (Amersham Pharmacia Biotech, Milan, Italy) by standard techniques and provided by the DBA Registries as de-identified material for further analyses.

Healthy controls (adult blood and cord blood samples) used for the CD34+ cell expansion studies were published in Federici et al. (2019).

### Polymorphism Determinations

Genotyping was performed for the following nine SNPs of the *NR3C1* gene: rs6198, rs6196, rs10482616, rs860457, rs7701443, rs3338, rs33389, rs6190, and rs10482605. The SNPs rs6196, rs10482616, rs860457, rs7701443, rs3338, and rs33389 were analyzed through PCR with allelic discrimination using the Taqman SNP genotyping assay (Applied Biosystems™, Waltham, Massachusetts, United States) as described by the manufacturer. Genotyping for rs6190 and rs10482605 was performed by restriction fragment lengths polymorphism analysis, employing the primers CTCAGCTGTGCAAATGGATTG (forward) and TCACAGTAGCTCCTCCTCTTAG (reverse) for rs6190, and GAGCTCCCGAGTGGGTCT (forward) and AACCTGTTGGTGACGCTTG (reverse) for rs10482605. For allelic discrimination, the amplified PCR products were subsequently digested with the restriction enzymes HpyAV (rs6190) and BslI (rs10482605). Results of the genotyping in individual DBA patients and healthy controls are detailed in Supplementary Tables S2 and S3. All SNPs were in Hardy-Weinberg equilibrium in both DBA and control group (*p*>0.05, data not shown).

### *Ex vivo* Expansion of Erythroid Cells and Reverse-Phase Protein Array

CD14negCD34pos cells (10^4^cells/ml) from three adult blood (AB1, AB2, and AB3) and three cord blood (CB1, CB2, and CB3) were cultured in parallel for 10days in cultures stimulated with SCF (100ng/ml, Amgen, Thousand Oaks, CA, United States), EPO (3U/ml, Janssen, Raritan, NJ, United States), IL-3 (10ng/ml, RD System, Minneapolis, MN, United States) with and without dexamethasone (Dex, 10^−6^ M; Sigma; BCMD). At day 10, cell numbers and viability were assessed by microscopic evaluation after trypan blue staining (Boston Bioproducts, Ashland, MA, United States), and phenotypic analysis was performed by flow-cytometry using Fluorescein Thiocyanate (FITC)–conjugated CD36 and phycoerythrin (PE)–conjugated CD235a, as described or appropriate isotype controls (all from Becton Dickinson Biosciences, Franklin Lakes, NJ, United States). Fluorescence intensities were measured with FACS ARIA (Becton Dickinson Biosciences; Falchi et al., 2015; Federici et al., 2019). The cells were then subjected to reverse-phase protein array (RPPA) analyses as described (Federici et al., 2019). Internal standardization was performed using the software package JMP v6 (SAS Institute, Cary, NC, United States), two-way hierarchical clustering was performed with the Wards method and significance was evaluated by two-group Wilcoxon test (significance cut off *p*≤0.05). All the data sets are available at http://capmm.gmu.edu/data. The data obtained with cells expanded without Dex are unpublished while those obtained with cells expanded with Dex have been the subject of a previous publication (Federici et al., 2019) and are reported here for comparison.

### Statistical Analyses

The association of specific markers in DBA was evaluated by several statistical methods implemented using the Plink tool.^1^ Firstly, the univariate logistic regression (by plink-logistic option) was adopted to calculate the odds ratio (OR) and 95% CI under the additive and the dominant model. The additive model assigned “0” for homozygotes of major allele (MM), “1” for heterozygotes of major allele (Mm), and “2” for homozygotes of minor allele (mm). Differently, the dominant model an assigned “0” for MM and “1” for both Mm and mm. In addition to the logistic regression analysis, the association between SNP and the disease was also evaluated adopting the chi-squared statistic (available with plink – model option). In particular, this is a multiple analysis approach that test simultaneously five different association models: “Allelic” which compares frequencies of alleles in cases vs. controls (M vs. m), “Cochran-Armitage trend test” similar to “Allelic” without assuming the HW Equilibrium (M vs. m), “Genotypic” which provides a general test of association in the 2-by-3 table of disease-by-genotype (MM vs. Mm vs. mm), Recessive (mm vs. Mm+MM), and Dominant (MM vs. Mm+mm) both designed to test the association of the minor allele. The comparative evaluation among the different models allows to better understand which type of association underlies the data distribution. Results from *ex vivo* cultures are expressed as Mean (±SD) and significance levels (*p*<0.05) analyzed by paired *t* test.

## Results

### Clinical Characteristics of Cohort of DBA Patients Included in the Study

Demographic, causal mutations and disease manifestation (GR responsiveness and age of anemia onset) are summarized in Table 1. The patients are 60% females and 40% males. Causal mutations are known for 73 of the 91 DBA patients investigated and included RPL35a (*n*=2), RPL5 (*n*=17), RPL11 (*n*=10); RPS26 (*n*=12), RPS19 (*n*=31), and RPS17 (*n*=1) for a total of 29 patients carrying mutations for L protein genes and 44 ones for S protein genes. Eight patients had no known mutations. Treatment outcome is known for 81 of the 91 patients: 36 (40%) of them responded and 45 (49%) did not respond to glucocorticoids. Age of disease manifestation is known for 79/91 patients: 55 displayed the disease before and 24 after 4months, with an average age at diagnosis for the all cohort of 79 patients of 6.8±15months.

**Table 1:** Demographic and clinical data of the Diamond Blackfan anemia (DBA) patients and healthy controls included in the study.

Baseline characteristics	Diamond-Blackfan anemia	Healthy
*n* (%)	91 (71%)	37 (29%)
Gender (*n*, % of total)	78 (86%)	n.a
Female	47 (60% of 78)	
Male	31 (40% of 78)	
Sources (*n*, %)		
Italy	65 (71%)	22 (32%)
France	18 (20%)	5 (14%)
Check	8 (9%)	
USA		10 (27%)
Mutation status (*n*, %)		
Unknown	18 (20%)	-
Mutation in S genes	44 (48%)	-
Mutation in L genes	29 (32%)	-
Treated (*n*, % of total)		
Unknown	10 (11%)	-
Yes	81 (89%)	-
No	-	37 (100%)
Response (*n*, % of treated)		
Yes	36 (yes and remission; 44%)	-
No	45 (no and dead; 56%)	-
Diagnosis at 4months (*n*, %)		
Known	79 (87% of total)	-
Before	54 (59% of known)	-
After	25 (28% of known)	-

### Comparison of the Frequency of the *NR3C1* SNPs Between DBA and Healthy Controls

The minor allele frequencies of the nine *NR3C1* SNPs investigated in DBA and healthy controls are summarized in Table 2 while the frequency of eight of them is compared in Table 3. To avoid biases due to the low number and unmatched ethnicity of healthy controls (several healthy controls were from the US of unknown ethnicity, while DBA patients are all European, Table 1) analyzed in parallel, the frequencies were also compared with the minor allele frequencies described in available genetic data bases of the Italian population included in the Network for the Italian Genome (*n*=1,686; http://nigdb.cineca.it) and of Italian (mainly from Tuscany) and European populations available in the International Genome Sample Resource (IGSR), a catalog of human genetic variations built by the 1,000 Genomes Project (*n*=522; https://www.internationalgenome.org/). Due to low amounts of DNA available for the study, all the nine SNPs were analyzed only in 46 (51%) of the patients and in eight (22%) of the healthy controls. Although, more than 89% of the patients and 65% of the healthy controls had more than five SNPs analyzed, we recognize that this technical limitation may have decreased the power of the statistical analyses.

**Table 2:** Summary of the single nucleotide polymorphisms (SNPs) investigated and of their frequency in the DBA population, in healthy controls and in publicly available data bases of the Italian and European populations.

			Frequency of Minor Allele								
SNP	Location	Alleles Major/Minor	DBA	Healthy controls	*p* value DBA vs. healthy controls	Tuscany populatio*n* (TSI)	*p* value DBA vs. TSI	Italian population (NIG)	*p* value DBA vs. NIG	European population	*p* value DBA vs. European population
rs6198	exon 9	A/G	0.2262 (*n* =84)	0.1 (*n* =15)	0.116	0.2297	0.9343	-	-	0.17	0.09866
rs6196	exon 9	A/G	0.1402 (*n* =82)	0.08333 (*n* =24)	0.298	0.1306	0.7845	0.0883	**0.02368**	0.15	0.6435
rs860457	intron 4	T/C	0.375 (*n* =88)	0.1875 (*n* =24)	**0.015**	0.3559	0.6935	-	-	0.33	0.1989
rs33388	intron 2	A/T	0.3696 (*n* =69)	0.525 (*n* =20)	0.078	0.3829	0.8	-	-	0.45	0.08362
rs33389	intron 2	C/T	0.1308 (*n* =65)	0.04762 (*n* =21)	0.135	0.1261	0.8998	-	-	0.15	0.5346
rs6190	exon 2	G/C	0 (*n* =76)	0 (*n* =15)	NA	0.0180	0.09615	0.0196	0.08164	0.03	**0.03429**
rs10482616	intron 1	G/A	0.08621 (*n* =87)	0.2083 (*n* =24)	**0.018**	0.1441	0.07685	-	-	0.13	0.1092
rs10482605	exon 1C	C/T	0.1267 (*n* =75)	0.125 (*n* =12)	0.982	0.2387	**0.00728**	-	-	0.18	0.1307
rs7701443	intron 1	A/G	0.4277 (*n* =83)	0.4583 (*n* =24)	0.706	0.3423	0.08634	-	-	0.39	0.3067

Value of p refers to the chi-squared allelic association test. Statistically significant (*p*<0.05) *p* values are indicated in red fonts.

**Table 3:** Statistical analyses with logistic regression (assuming either additive or dominant effect of the minor allele) of the SNPs in DBA and healthy controls, responders vs. non responders and age to disease manifestation before 4months.

	Cases vs. Healthy controls						Responders vs. Non-Responders						Diagnosis at 4months					
	Additive model			Dominant model			Additive model			Dominant model			Additive model			Dominant model		
SNP	OR	95% CI	*p* value	OR	95% CI	*p* value	OR	95% CI	*p* value	OR	95% CI	*p* value	OR	95% CI	*p* value	OR	95% CI	*p* value
rs6198	2.56	0.74–8.89	0.138	2.59	0.68–9.87	0.164	1.15	0.53–2.49	0.718	1.14	0.45–2.90	0.777	1.52	0.68–3.40	0.309	1.59	0.60–4.18	0.351
rs6196	1.86	0.59–5.84	0.289	1.83	0.56–5.96	0.314	0.60	0.21–1.71	0.335	0.63	0.20–1.91	0.410	3.76	1.01–13.92	**0.048**	3.83	1.01–14.59	**0.049**
rs860457	2.82	1.23–6.48	**0.014**	2.78	1.09–7.06	**0.032**	1.11	0.56–2.18	0.769	1.32	0.51–3.37	0.567	2.43	1.09–5.43	**0.030**	2.60	0.97–6.99	0.058
rs33388	0.53	0.26–1.09	0.086	0.71	0.24–2.07	0.530	0.86	0.40–1.85	0.698	0.79	0.29–2.17	0.645	0.64	0.27–1.52	0.313	0.64	0.20–2.02	0.450
rs33389	3.05	0.66–14.04	0.151	3.10	0.65–14.80	0.156	0.63	0.20–2.00	0.432	0.68	0.19–2.40	0.551	2.64	0.55–12.64	0.226	2.66	0.52–13.63	0.242
rs10482616	0.33	0.13–0.85	**0.021**	0.35	0.13–0.94	**0.037**	2.25	0.66–7.64	0.193	2.25	0.66–7.64	0.193	0.37	0.11–1.25	0.109	0.37	0.12–1.25	0.109
rs10482605	1.01	0.29–3.59	0.982	0.88	0.21–3.62	0.859	1.13	0.42–3.00	0.809	1.17	0.36–3.80	0.798	1.63	0.52–5.07	0.400	1.45	0.39–5.35	0.576
rs7701443	0.90	0.49–1.64	0.725	0.88	0.34–2.31	0.800	0.99	0.53–1.86	0.982	0.80	0.31–2.10	0.656	0.74	0.39–1.38	0.340	0.85	0.32–2.26	0.741

OR, odds ratios;

CI, confidence interval.

Values of *p*<0.05, giving significant association, are indicated in red.

All the SNPs were further tested by the chi-squared statistic under several models (see Supplementary Table S3).

Cases vs. control analyses suggest that the frequency of the minor allele of rs860457 (increased, *p*=0.015) and rs10482616 (decreased, *p*=0.018) is altered in DBA patients with respect to healthy controls, while that of rs10482605 is increased with respect to the Tuscany registry (*p*=0.0073), that of s6196 is decreased with respect to the Italian registry (*p*=0.024) and that of rs6190 is increased with respect to the European registry (*p*=0.034). The association of the SNPs rs860457 and rs10482616 with the disease was confirmed by further model-based tests that revealed a statistically significant association according to the Genotypic, Cochran-Armitage, Allelic, and Dominant models and Genotypic and Recessive models (Table 3; Supplementary Table S4).

### Correlation Between the Frequency of the *NR3C1* SNPs and Disease Manifestation in DBA

A recent study describing the long-term experience of the Italian DBA registry indicated that mutations in L genes have a more severe disease manifestation than patients carrying mutations in S genes (Quarello et al., 2020). We were inspired by this observation to compare the association of L and S mutations with disease manifestation and/or the *NR3C1* SNPs. This preliminary analysis indicated that mutations in L and S genes were not associated with *NR3C1* SNPs. In addition, causal mutations (not gender) were neither associated with glucocorticoid response nor with age of disease manifestation (data not shown) in this small cohort of patients.

Logistic regression in both additive and dominant models was performed to assess the association of single SNPs with DBA (cases vs. controls), treatment (responders vs. non-responders), and time of diagnosis (DBA patients diagnosed before or after 4months; Table 3).

None of the SNPs met the threshold of significance of 5% in the response vs. nonresponsive groups (Table 3). By contrast, the minor allele of the SNPs rs6196 and rs860457 (both increased, *p*=0.048 and *p*=0.03) met the threshold criteria in the age of disease manifestation analysis (diagnosis before 4months; Tables 3 and 4) according to the additive model. With regard to disease manifestation, association of these SNP was also evident with the dominant (rs6196 only), Cochran-Armitage, and Allelic (both rs6196 and rs860457) models (Supplementary Table S4).

**Table 4:** List of endpoints of the apoptosis pathway included in the RPPA array and of their fold changes in erythroid cells expanded with and without Dex either from adult blood or cord blood.

Gene ID	Protein	Adult Blood		Cord Blood	
		FC -Dex/+Dex	Prob>ChiSq	FC -Dex/+Dex	Prob>ChiSq
AKT1	AKT (T308)	0.421311199	0.268285884	0.69198831	0.51269076
BAD	Bad	0.638262161	0.126630458	1.053929365	0.827259347
BAD	Bad (S112)	0.856291541	0.51269076	0.941886938	0.827259347
BAD	Bad (S136)	1.133089531	0.126630458	1.099132457	0.51269076
BAD	Bad (S155)	0.77804854	0.275233524	1.065222961	0.51269076
BAK	Bak	1.69288473	0.126630458	0.912686031	0.275233524
BAX	Bax	1.503605762	**0.049534613**	1.105640833	0.275233524
BCL2	Bcl-2	1.384055823	0.51269076	0.988528746	0.827259347
BCL2	Bcl-2 (S70)	1.083327952	0.275233524	0.960573233	0.275233524
BCL2	Bcl-2 (T56)	0.807736537	**0.049534613**	1.169746052	0.184038627
BCL2L1	Bcl-xL	/	/	/	/
CASP3	cl Caspase 3 (D175)	5.536026973	0.275233524	0.214693893	0.275233524
CASP6	cl Caspase 6 (D162)	2.29166442	0.827259347	0.789668893	0.275233524
CASP7	cl Caspase 7 (D198)	32.19465592	**0.049534613**	2.279410458	0.275233524
CASP9	cl Caspase 9 (D315)	1.085763655	0.827259347	0.599717596	0.51269076
CYCS	Cytochrome C	1.888577547	**0.049534613**	1.02653214	0.827259347
FADD	FADD (S194)	0.286144187	**0.049534613**	0.723546381	0.51269076
NFKBIA	IkB alpha (S32/36)	1.262011985	**0.046301595**	0.917847497	0.275233524
TP53	/	/	/	/	/
PRKACA	PKA C (T197)	1.190717477	0.275233524	0.988244428	0.51269076
XIAP	XIAP	0.510740869	0.275233524	4.97441997	0.121183273

Hits that met the threshold for upregulation (Fold Change<0.5) and downregulation (Fold Change>2) are in red and in green fonts, respectively. Statistically significant *p* values (*p*<0.05 by Wilcoxon test) are shown in bold (For further details see: http://capmm.gmu.edu/data).

### GR Activation Induces a More Pronounced Ribosomal Signature in Erythroid Cells Expanded From Cord Blood Than in Those Expanded From Adult Blood

In humans, the major erythropoietic site switches from the fetal liver to the bone marrow at birth (Papayannopoulou and Migliaccio, 2018). This switch is associated with deep changes in the growth properties and type of haemoglobin expressed by erythroid progenitor and precursor cells (Papayannopoulou and Migliaccio, 2018). To undergo this profound reprogramming, the erythroid progenitor cells take a pause from active differentiation as reflected by the transient anemia of the newborns which last for few months after birth. We hypothesized that the mechanisms which underlay the association between the SNPs of GR and time to disease onset in DBA may be related to the process with which erythroid progenitor cells change their sensitivity to GR activation at birth. To test this hypothesis, we compared the *ex vivo* expansion and phosphoproteomic profiling of erythroid cells expanded in parallel cultures with and without Dex by three adult blood and three cord blood.

**Figure 1:**
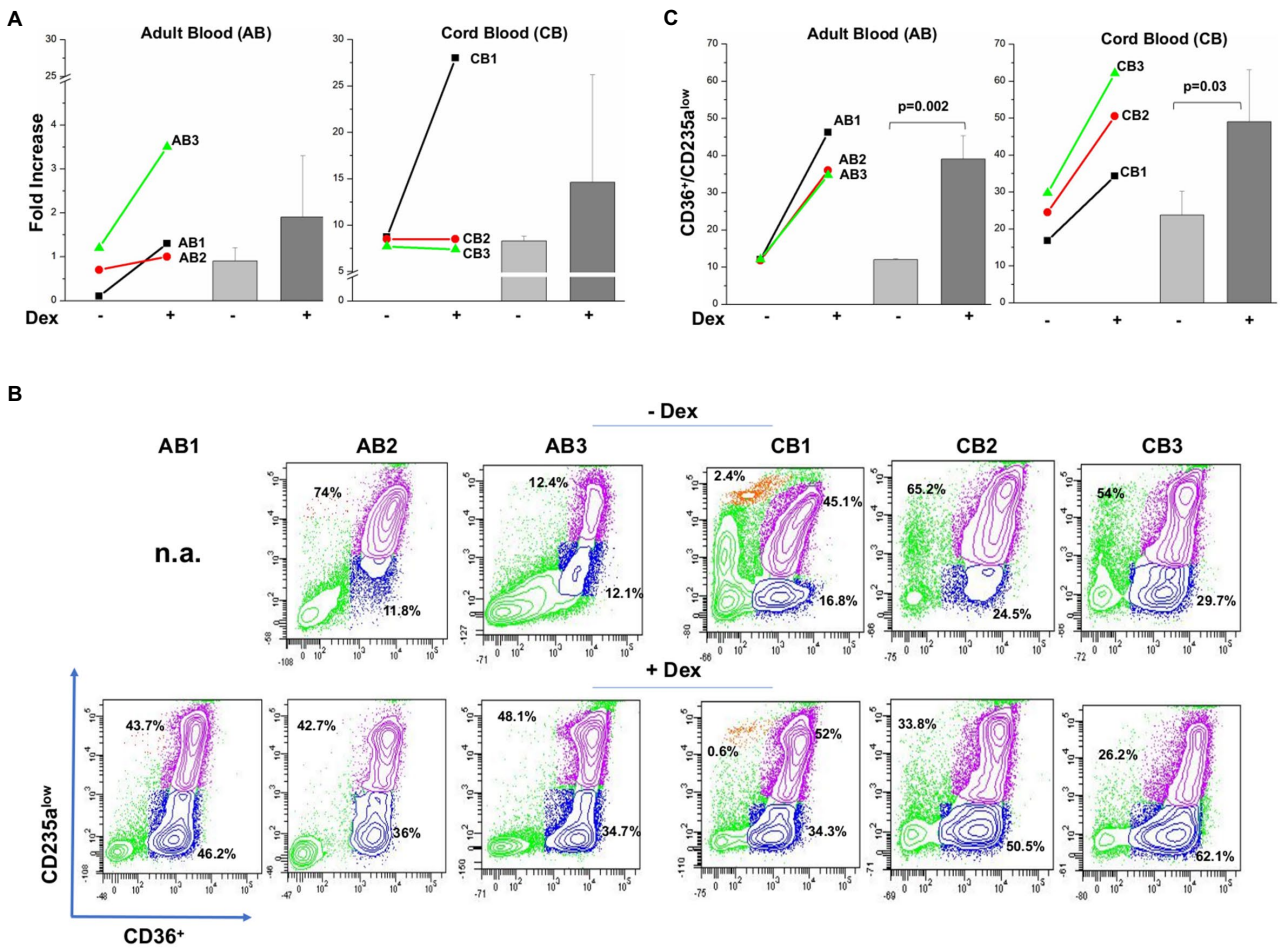
Both adult blood and cord blood generate by day 10, a greater proportion of immature erythroid cells in cultures with Dex than in those without Dex. **(A)** Fold increase of erythroid cells generated by day 10 in cultures of three separate adult blood (AB1, AB2, and AB3) and cord blood (CB1, CB2, and CB3) with and without Dex. Values are presented as individual values for each donor and as Mean (±SD). **(B)** Flow cytometry analyses for CD63 and CD235a of the cells present at day 10 in the cultures presented in **(A)**. These cells were subjected to the phosphoproteomic analyses presented in Figure 2. CD36/CD235a expression classifies the cultured cells into immature (blue contour) and matures (purple contour) erythroblasts (Falchi et al., 2015; Federici et al., 2019). The frequency of the immature and mature cells obtained from each source is indicated within the quadrant. The profile of the cells obtained with Dex has been already reported (Federici et al., 2019). **(C)** Frequency of immature erythroid cells (blue gate in **B**) present at day 10 in culture of adult blood and cord blood stimulated with and without Dex. Values are presented as individual values for each donor and as Mean (±SD). Statistical analysis was performed by paired *t* Test.

As expected (Migliaccio et al., 2002; Ashley et al., 2020), by day 10, adult blood generated on average, twice more erythroid cells in cultures with Dex than in those without Dex (0.7±0.6 vs. 1.9±1.4; Figure 1A), although due to the great variability and low number of donors analyzed, these experiments do not have the power to reach statistical significance. On average, cord blood generates greater numbers of erythroid cells than adult blood. However, in this case, only one donor generated more cells with Dex than without Dex (Figure 1A). Dex has been hypothesized to increase the erythroid output in culture by retaining the cells immature and capable to proliferate (Migliaccio et al., 2011; Ashley et al., 2020). It is then a surprise, that, although increased cellular outputs are observed only with adult blood, a greater proportion of cells from both sources are immature in the presence of Dex (CD36+/CD235aneg cells: 12.0%±0.2 vs. 39.0%±6.3, *p*=0.002, and 23.7%±6.5 vs. 49.0%±14.0, *p*=0.03 in cultures with and without Dex from adult blood and cord blood, respectively).

To obtain mechanistic insights on the effects of Dex on cells from adult blood and cord blood, the erythroid cells from the two sources were subjected to RPPA analyses. Hit points which met significant levels of fold change threshold are summarized in Supplementary Tables S5 and S6 for adult blood and cord blood, respectively. For adult blood, 22 hits were significantly regulated by Dex (13 upregulated and nine downregulated). For cord blood, only seven hits were significantly regulated by Dex (four upregulated and three downregulated). Only three hits were regulated by Dex in both samples (STAT3 downregulated in both) and CFL1 and FOXO1/O3 (both downregulated in adult blood and upregulated in cord blood). The fact that in adult blood downregulation of FOXO1/O3 is associated with upregulation of AKT(T308) is consistent with the report that AKT(T308) is responsible for decreasing FOXO1/O3 activity in murine adult hematopoietic stem/progenitor cells (Liang et al., 2016).

**Figure 2:**
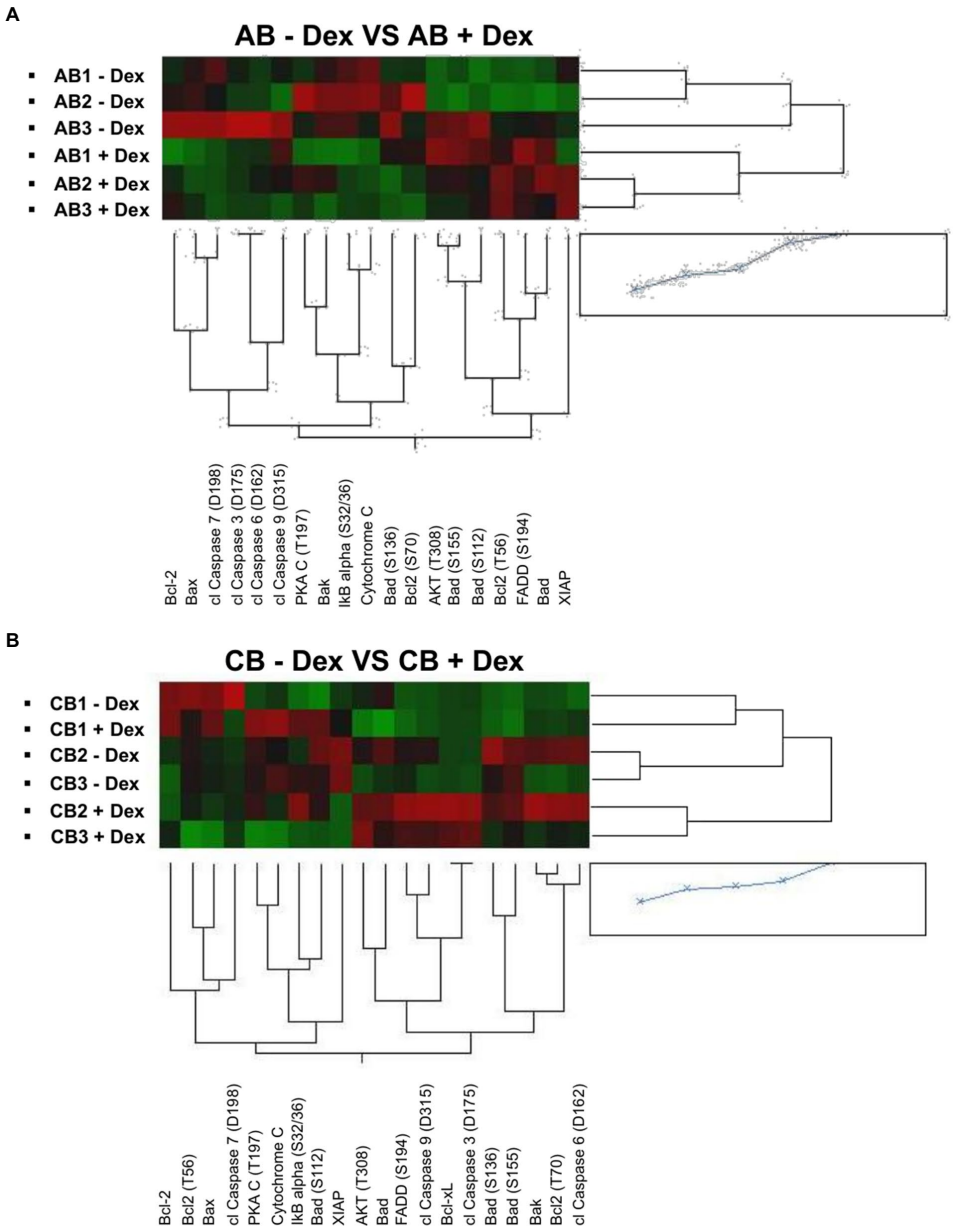
Dex activates the pro-apoptotic signal in cells from adult blood but not in those from cord blood. Heatmaps comparing reverse-phase protein array (RPPA) analysis of protein lysates of erythroid cells from the three adult blood **(A)** and three cord blood donors (**B**; identified with the same code used in Figure 1).

By morphological analyses, we have previously identified that in cultures of adult blood supplemented with Dex, the positive effects resulting from the induction of self-replication are restrained by the fact that Dex also increases cell death (Migliaccio et al., 2011). To identify changes in protein phosphorylation which could explain the different effects of Dex on *ex vivo* expansion of adult blood and cord blood, we analyzed in detail hits within the apoptotic pathway. Hierarchical clustering of the proteins of this pathway of adult blood revealed a good sample segregation between cells cultured with Dex and without Dex (Figure 2A). Pathway analyses of adult blood indicates that two hits (FADD, upregulated) and CASP7 (downregulated) meet both the threshold and significance criteria for events regulated by Dex with four additional significant hits (BAX, CYCS, and NFKBIA, upregulated, and BCL2, downregulated) barely missing the threshold (Table 4). These results confirm the morphological observation indicating that in adult blood Dex induced apoptosis.

By contrast, the apoptotic profile of cord blood samples is not only partially different from that of adult blood but also displays inconsistent segregation among samples (Figure 2B). CB1 express a similar profile when cultured with and without Dex while CB2 and CB3 expressed a different, although consistent, profile under the two conditions. As a result, analyses of the apoptotic pathway of cord blood did not indicate any hits that meets the threshold and significance criteria among samples with and without Dex (Table 4).

Pathway analyses for proteins involved in ribosomal activity of cord blood identified that Dex significantly increase hits that promote mRNA binding to 40S [eIF4G(S1108); Bohlen et al., 2020], [FC 0.24, *p*=0.049 and S6KA1(S235/236); Rosner et al., 2011; 0.25, *p*=0.045], and facilitate mRNA translation [mTOR (S2448), FC=0.57, *p*=0.049; Morita et al., 2015; Table 5]. By contrast, Dex significantly increased hits that inhibit initiation [RSK3 (T356/S360), FC=2.18, *p*=0.045; Jagilinki et al., 2016] or inhibit mRNA translation (DEPTOR, FC=0.66, *p*=0.045; Catena and Fanciulli, 2017). Although it barely missed the threshold, only MSK1 (S360; Wiersma et al., 2016), a hit which favor the initiation process, is significantly decreased by Dex in cells from both cord blood and adult blood. These results suggest that Dex promote protein translation in cells from cord blood but not in those from adult blood.

**Table 5:** Ribosomal signature in erythroblasts obtained with and without Dex from Adult Blood and Cord Blood.

			Adult Blood		Cord Blood	
Activity	Gene ID	Protein	-Dex/+Dex Fold Change	Prob>ChiSq	-Dex/+Dex Fold Change	Prob>ChiSq
mRNA translation						
Binding to 40S (Bohlen et al., 2020)	EIFG41	eIF4G (S1108)			**0.24**	**0.0495**
Initiation (Rosner et al., 2011; Jagilinki et al., 2016; Musa et al., 2016; Wiersma et al., 2016)	*EIF4EBP1*	4E-BP1 (S65)	**0.36**	0.2752		
	*RPS6KA1*	Ribosomal Protein S6KA1 (S235/236)			**0.25**	**0.0495**
	*RPS6KA2*	Ribosomal Protein RSK3 (T356/S360)	**2.18**	**0.0495**		
	*RPS6KA5*	Ribosomal Protein MSK1 (S360)	**1.90**	**0.0495**	**1.30**	**0.0495**
Regulators of Translation						
Positive (Morita et al., 2015)	*MTOR*	mTOR (S2448)			**0.57**	**0.0495**
Negative (Catena and Fanciulli, 2017)	*DEPTOR*	*DEPTOR*	**0.66**	**0.0495**		

Hits which meet the threshold for upregulated (Fold Change<0.5) and downregulated (Fold Change>2) are in red and in green fonts, respectively. Statistically significant *p* values (*p*<0.05 by Wilcoxon test) are shown in bold (For further details see: http://capmm.gmu.edu/data).

## Discussion

In this study, we used PCR single-strand conformation polymorphism analyses to determine the frequency of eight clinically relevant *NR3C1* SNPs in a cohort of 91 DBA patients, 65 from the Italian Registry, and 26 from other European registries. A small cohort of healthy volunteers (*n*=37) was analyzed in parallel as technical control. The study has strengths as well as intrinsic limitations. Although for a rare disease such DBA, our patient sample size is strength, in the context of robust analyses for genetic association, the sample size was small. Other limitations which may have weakened the power of the statistical analyses include incomplete patient demographic information for some patients, and some SNP genotypes were unavailable. However, the data presented here should be useful in improving our understanding the pathobiology of DBA and strongly support the need for further investigation on the effects of genetic variability in the manifestation of this disease.

The results of the DBA patients were compared with frequencies reported in public genetic data based on the Italian and European populations. Although we found that the minor allele frequency of some of the SNPs was different between DBA and the normal populations, the differences were not consistent among data bases, indicating that overall the frequency of these minor alleles in DBA is not different than in the normal population.

Although the SNPs were not correlated neither with gender nor with the driver mutation (L or S), the lack of association with the driver mutation is particularly intriguing and may have been affected by the insufficient power of the study. When the frequency of the minor alleles was correlated with disease manifestation, we did not find any correlation with glucocorticoid response, suggesting that in DBA, by contrast with autoimmune-diseases, polymorphism of *NR3C1* is not a major driver in determining the response to glucocorticoids. To a surprise, we found a significant correlation between rs6196 and rs860457 and time to diagnosis greater than 4months (Table 6). Since the DBA is a disease which manifests itself mostly after birth, we hypothesize that these SNPs may delay disease onset in DBA because they regulate the response of erythroid cells to glucocorticoids during ontogeny.

**Table 6:** Summary of the SNPs investigated, of their association with diagnosis at 4months in DBA, of their published biological effects and of their association with other diseases.

SNP	Location	Alleles Major/Minor	Frequency of the minor alleles in patients with diagnosis < vs. > 4months	*p* value^*^	Biological effects	Disease/Phenotype
rs6198	exon 9	A/G	0.2596 vs. 0.1852	0.309	Increased expression of GRβ	Rheumatoid arthritis; Systemic lupus erythematosus; Reduced central adiposity in women; Diamond Blackfan anemia; Polycythemia Vera; Reduced diabetes risk in patients with cushing’s syndrome;
**rs6196**	**exon 9**	**A/G**	**0.1765 vs. 0.0577**	**0.048**	**Unknown**	**Corticoid resistance in children with Crohn’s Disease**
**rs860457**	**intron 4**	**T/C**	**0.4412 vs. 0.2600**	**0.030**	**Unknown**	**Corticoid resistance in children with Crohn’s Disease**
rs33388	intron 2	A/T	0.3375 vs. 0.4250	0.313	Increased expression of GR*γ*	Increased sensitivity to corticosteroids
rs33389	intron 2	G/A	0.1500 vs. 0.0625	0.226	Increased expression of GR*γ*	Increased sensitivity to corticosteroids
rs6189/rs6190	exon 2	A/GG/A	0.0000 vs. 0.0000	Na	Unknown	Major depression; Rheumatois arthritis; Corticoid resistance in children with Crohn’s Disease
rs10482616	intron 1	G/A	0.0600 vs. 0.1346	0.109	Unknown	Corticoid resistance in children with Crohn’s Disease
rs10482605	exon 1C	C/T	0.1375 vs. 0.0833	0.400	Decreases levels of GRα	GCs resistance in major depression
rs7701443	intron 1	A/G	0.3942 vs. 0.4808	0.340	Unknown	Corticoid resistance in children with Crohn’s Disease

* Logistic regression under additive model. Minor alleles with known positive and negative effects on other disease are indicated in red and green fonts, respectively. SNPs significantly correlated with time of diagnosis are in red font.

The literature on the response of erythroid cells to glucocorticoids during ontogeny is controversial. The first evidence that erythroid cells from different neonates may display different response to glucocorticoids was provided by Papayannopoulou and colleagues who demonstrated that Dex accelerates the spontaneous γ to β switch occurring in the two MEL hybrid model containing the human chromosome 11 from fetal liver in culture (Papayannopoulou et al., 1986; Zitnik et al., 1995). This model is composed by murine erythroleukemic cell lines fused with erythroid cells from the fetal liver. The fused murine cells randomly lose all but one of the human chromosomes, allowing to select clones which specifically retain chromosome 11. This paper not only indicated for the first time that steroids have a direct effect on globin gene switching but also observed great variability in the speed of the switch among hybrid clones containing chromosomes derived from different donors, with some clones switching in few months and others switching after >6months of culture.

Our laboratory has demonstrated that CD34^+^ cells from cord blood respond more readily than those from adult blood to glucocorticoids in culture generating more erythroid cells over time than the other ones (Federici et al., 2019). These results suggest that GR is more active in cord blood cells than in the adult ones. In human, alternative splicing induces the formation of at least two forms of GR binding subunits. The most studied of these forms is GRα, that upon binding to its ligand, migrates to the nucleus where it activates/suppresses expression of target genes (Zhou and Cidlowski, 2005). However, an alternative splicing between exons 3 and 4 generates GRγ, an isoform containing an additional amino acid (Arg) in the DNA-binding domain which halves the transactivation potential of the protein (Rivers et al., 2009). In previous publications, we identified that GRα is expressed by erythroid cells expanded both from adult blood and cord blood (with cells from cord blood expressing on average more GRα than those from adult blood) and that in adult blood, reduced expression of GRα is partially compensated by expression of GRγ, the less active isoform which is not expressed in cells from cord blood (Migliaccio et al., 2012). These results suggest that, a switch from a GRα to a GRα+GRγ expressing state reduces the response of erythroid cells to glucocorticoids after birth.

In addition to quantitative differences in glucocorticoid response, erythroid cells from cord blood and adult blood also express qualitative differences. This hypothesis is supported by observations indicating that transcriptional activity of GR is strictly dependent on the chromatin configuration of targeted cells (Biddie et al., 2011; Wiench et al., 2011) and that the chromatin configuration of erythroid cells is deeply altered during ontogeny (McKinney-Freeman et al., 2012). We tested this hypothesis by comparing the phosphoproteomic profiling of erythroid cells generated *in vitro* with or without the GR agonist Dex by CD34^+^ cells from adult blood and cord blood. Our results indicate that, as reported for adult muscle cells (Wang et al., 2006); Dex inhibits initiation of mRNA translation and protein biosynthesis in adult erythroid cells. By contrast, however, Dex induces a series of events which indicate activation of ribosome activity in erythroid cells from cord blood. Furthermore, pathway analyses for proteins involved in apoptosis of these phosphoproteomic profilings indicate that Dex induces a pro-apoptotic state in adult cells but not in cells from cord blood. These results indicate that GR is likely to exert qualitatively different effects in the erythroid cells from the two sources, with more positive effects on mRNA translation in cord blood and promoting apoptosis in. It is possible that the positive effects of GR on ribosomal functions in cord blood may protect DBA patients from expressing anemia before birth.

By contrast with our previous studies obtained with cord blood (Federici et al., 2019), recently published data using CD34+ cells from cord blood (and unpublishes data from the Da Costa laboratory) indicate that these cells do not respond to glucocorticoids (Ashley et al., 2020). The data presented in this manuscript indicate instead that the response to Dex of different cord blood donors is highly heterogenous with some of them appearing as glucocorticoids unresponsive in terms of fold expansion. Since the expansion of cord blood in the absence of Dex is so much greater of that of adult blood, it is possible that some cord blood may appear Dex unresponsive *in vitro* because they express maximal response already to the physiological levels of glucocorticoids present in the fetal bovine serum. We hypothesize here that, as suggested by the Papayannopoulou laboratory (Papayannopoulou et al., 1986; Zitnik et al., 1995), the response of erythroid cells to glucocorticoids changes not only qualitatively but also quantitatively at birth from a hyper-responsive to a low-responsive state. The mechanism which underlays this switch in glucocorticoid response may or may not be correlated with that determining the hemoglobin switching but it is likely to be genetically regulated. In this regard, it is very interesting that time to disease diagnosis was found to be correlated with two SNPs, rs6196, and rs860457, the biological functions of which is still unknown (Table 6) that have a configuration consistent with that of potential regulatory elements. rs6196 is a Synonymous Variant^2^ and a regulatory region variant because it is located in a region flanking a promoter.^3^ rs860457 is an Intron Variant^4^ and is in a region the function of which is still unknown.^5^ Additional studies, outside the purpose of this paper, are required to clarify whether these two SNPs regulates the cellular ability to respond to glucocorticoids during ontogeny by modulating the level and/or GR isoforms expressed by the cells.

In conclusion, we have identified two SNPs, rs6196 and rs860457, enriched in patients manifesting the disease before 4months. The fact that the configuration of these SNPs is consistent with that of regulatory regions, suggests the hypothesis, to be confirm by additional study with a large cohort of patients, that they regulate changes in glucocorticoid response during ontogeny.

## Data Availability Statement

The datasets presented in this study can be found in online repositories. The names of the repository/repositories and accession number(s) can be found in the article/**Supplementary Material**.

## Ethics Statement

The studies involving human participants were reviewed and approved by DNA from DBA patients was provided before 2010 and has been the subject of previous publications (Varricchio et al., 2011; Horos et al., 2012). Since the determination of the SNPs is a research on human biological samples (DNA) already collected and stored in biobanks (and not related to patients in the Provinces of Bologna and Ferrara), the chair of the Independent Ethics Committee of Area Vasta Emilia Centro (CE-AVEC) of the Emilia-Romagna Region, which supervises research conducted at the University of Bologna) declared in a Memo on 06/12/2018 not necessary to submit a request for opinion from the committee in order to perform the genomic testing. For studies on correlation between SNPs and disease manifestation, clinical information were provided to the University of Bologna by the University of Turin because patients or their parents signed an informed consent to share clinical data and DNA for clinical and research purposes as specified in the Local EC that authorized data collection and the possibility of sharing anonymous data (aut. N. 0105777 of the 31 October 2016). Healthy controls (adult blood and cord blood samples) used for the CD34+ cell expansion studies were published in Federici et al. (2019). Written informed consent to participate in this study was provided by the participants’ legal guardian/next of kin.

## Author Contributions

AL and VI revised the data and performed the statistical analyses. UR, LC, and DP provided DNA and clinical and genetic information from the patients from the Italian, French, and Czech registries. ID and LC revised the data and wrote the manuscript. AM designed the study, interpreted the data, and wrote the manuscript. All authors contributed to the article and approved the submitted version.

## Funding

This study was supported by grants from the National Cancer Institute (P01-CA108671), the Heart, Lung and Blood Institute (1R01-HL134684), and Associazione Italiana Ricerca Cancro (AIRC; IG23525).

## Conflict of Interest

The authors declare that the research was conducted in the absence of any commercial or financial relationships that could be construed as a potential conflict of interest.

## Publisher’s Note

All claims expressed in this article are solely those of the authors and do not necessarily represent those of their affiliated organizations, or those of the publisher, the editors and the reviewers. Any product that may be evaluated in this article, or claim that may be made by its manufacturer, is not guaranteed or endorsed by the publisher.

## Footnotes

^1^ https://www.cog-genomics.org/plink/1.9/

^2^ https://www.ncbi.nlm.nih.gov/snp/rs6196?vertical_tab=true#clinical_significance

^3^ https://www.ensembl.org/Homo_sapiens/Variation/Mappings?db=core;r=5:143281425-143282425;v=rs6196;vdb=variation;vf=164781822

^4^ https://www.ncbi.nlm.nih.gov/snp/rs860457?vertical_tab=true#publications

^5^ https://www.ensembl.org/Homo_sapiens/Variation/Explore?db=core;r=5:143308258-143309258;v=rs860457;vdb=variation;vf=165885502
