# Supplementary material for: The Glucocorticoid Receptor Polymorphism Landscape in Patients With Diamond Blackfan Anemia Reveals an Association Between Two Clinically Relevant Single Nucleotide Polymorphisms and Time to Diagnosis
Source: Front Physiol. 2021 Oct 13;12:745032. doi: 10.3389/fphys.2021.745032 (PMC8549833; doi:10.3389/fphys.2021.745032)
Supplement: Supplementary file 1 [file Data_Sheet_1.docx]

Supplementary Materials

Supplementary Tables

Table S1: Phenotypic and genotypic information of DBA patients.

| **Sources** | **Sample ID** | **Gene** | **Mutation** | **Time of diagnosis** | **Response** |
| --- | --- | --- | --- | --- | --- |
| Czech | # d1 | n.a. | n.a. | 1 year | yes |
| Czech | # d10 | n.a. | n.a. | birth | yes |
| Czech | # d11 | n.a. | n.a. | 3 months | no |
| Czech | # d2 | n.a. | n.a. | 2 months | no |
| Czech | # d3 | n.a. | n.a. | birth | yes |
| Czech | # d4 | n.a. | n.a. | > 1 year | unknown |
| Czech | # d6 | n.a. | n.a. | 1 year | no |
| Czech | # d9 | n.a. | n.a. | >1 year | unknown |
| France | # 168 | no known mutations | n.a. | unknown | yes |
| France | # 199 | S26 | CGH : Del Chr 12 : 56,426,191-56,438,551  12,361kb | 3 months | yes |
| France | # 215 | S19 | p.Arg62Trp | unknown | no |
| France | # 227 | no known mutations | n.a. | 4 months | no |
| France | # 240 | no known mutations | n.a. | 5 months | yes |
| France | # 254 | S19 | p.Arg62Trp | 2 months | no |
| France | # 262 | L5 | c.531_562del  p.Lys178GlyfsX3 | 1 month | no |
| France | # 303 | L35a | c.308 T>C  p.Val103Ala | 2 months | yes |
| France | # 35 | S19 | p.Arg62Gln | 3 months | no |
| France | # 36 | no known mutations | n.a. | 1 year 9 months | yes |
| France | # 367 | S19 | c.90delC | 1 month | yes |
| France | # 439 | L35a | CGH / Loss Chr 3 :  197.658.413-197.678.212  19,8kb | 3 months | no |
| France | # 50 | no known mutations | n.a. | 3 months | Dead |
| France | # 501 | L5 | c.1A>G p.Met1? | 2 months | no |
| France | # 587 | L11 | c.260T>C P.Leu87Pro | birth | yes |
| France | # 588 | L11 | c.164dup p.Tyr55X | birth | no |
| France | # 590 | L5 | c.637G>T p.Glu213X | 7 months | no |
| France | # 598 | L5 | c.73G>T p.Glu25X | birth | no |
| Italy | # 1035 | S19 | c.3G>A | 1 year 4 month | yes |
| Italy | # 1099 | S19 | c.412delG | 1 month | no |
| Italy | # 1239 | L11 | c.465_466delCA | 3 months | no |
| Italy | # 1240 | L5 | c.678C>A | 3 months 4 days | no |
| Italy | # 1241 | S19 | deleted | 2 months 8 days | yes |
| Italy | # 1252 | L11 | c.143_157+32del | 3 years 10 months | remission |
| Italy | # 1383 | L5 | c.283delT | 4 months | no |
| Italy | # 1389 | S19 | c.156G>C | 2 months 7 days | yes |
| Italy | # 1429 | S17 | deleted | 4 months 8 days | no |
| Italy | # 1448 | L5 | c.134_138delACACA | 1 month | yes |
| Italy | # 1459 | S19 | IVS1+1G>C | 4 months 21 days | yes |
| Italy | # 1460* | S19 | IVS1+1G>C | unknown | unknown |
| Italy | # 1466 | S19 | c.184C>T | 1 month 15 days | unknown |
| Italy | # 1491 | L5 | c.314_315delTT | birth | no |
| Italy | # 1504 | S19 | deleted | 1 month 4 days | no |
| Italy | # 1506 | L5 | c.324+5G>T | 1 year 11 months 5 days | remission |
| Italy | # 1508 | S19 | c.58delG | 1 month 8 days | yes |
| Italy | # 1518 | S26 | c.259C>T | birth | no |
| Italy | # 1529 | L5 | c.3G>C | 2 months | yes |
| Italy | # 1624 | no known mutations | n.a. | 1 month | no |
| Italy | # 1673 | no known mutations | n.a. | 2 months | remission |
| Italy | # 1676 | S19 | c.184C>T | 9 years | no |
| Italy | # 1703 | S19 | c.357-1G>T | 4 months 13 days | no |
| Italy | # 1707 | L5 | c.39_40insT | 1 month 9 days | yes |
| Italy | # 1722 | L11 | deleted | unknown | no |
| Italy | # 1816 | S19 | c.3G>T | 1 month | remission |
| Italy | # 1830 | L5 | c.172_173insA | 3 months | no |
| Italy | # 1904 | L11 | c.157+1_157+16del | 3 months 1 day | remission |
| Italy | # 1929 | S19 | c.384_385delAA | 4 months | unknown |
| Italy | # 1945 | S19 | c.301delC | 1 year 1 month | unknown |
| Italy | # 1981 | L11 | c.508-2A>G | unknown | no |
| Italy | # 2087 | S19 | c.185G>A | unknown | yes |
| Italy | # 2124 | L5 | c.175_176delGA | unknown | yes |
| Italy | # 2137 | S26 | c.50dupA | unknown | yes |
| Italy | # 2206 | S19 | c.288dupC | unknown | yes |
| Italy | # 2221 | S19 | deleted | unknown | no |
| Italy | # 2471 | L5 | c.535C>T | unknown | yes |
| Italy | # 272 | S26 | c.3+1G>C | 4 months 23 days | no |
| Italy | # 277 | S26 | c.4-1G>A | 1 month | Dead |
| Italy | # 279 | S26 | c.4-1G>A | unknown | unknown |
| Italy | # 284 | S26 | c.51delC | 2 months 23 days | no |
| Italy | # 293 | S19 | c.184C>T | birth | remission |
| Italy | # 359 | S19 | c.184C>T | 2 month 1 days | Dead |
| Italy | # 363 | no known mutations | n.a. | 3 years 9 month | remission |
| Italy | # 367 R | S19 | c.53_54insAGA | birth | unknown |
| Italy | # 383 | L5 | c.336delG | 2 years 8 months | no |
| Italy | # 387 | L11 | c.469delA | 1 month | dead |
| Italy | # 456* | no known mutations | n.a. | 2 months 1 day | yes |
| Italy | # 471 | S19 | c.302G>A | 3 months 13 days | remission |
| Italy | # 472 | S19 | c.302G>A | 4 months 13 days | yes |
| Italy | # 476 | S19 | t (1;19) | 1 month | no |
| Italy | # 477 | S19 | c.411+1G>A | birth | no |
| Italy | # 502 | S26 | c.222_225delTGTG | 11 months | remission |
| Italy | # 518 | S19 | c.184C>T | 3 months | remission |
| Italy | # 521 | L5 | c.169_172delAACA | 11 months 1 day | unknown |
| Italy | # 548* | S19 | c.3G>C | 2 months | no |
| Italy | # 647 | S19 | c.1-1G>A | 7 months 12 days | remission |
| Italy | # 667 | S26 | c.3+2T>A | 1 month 8 days | no |
| Italy | # 671 | L11 | c.95_96delGA | 2 months 7 days | no |
| Italy | # 672 | no known mutations | n.a. | 2 months 16 days | no |
| Italy | # 673 | L5 | c.189+1G>A | 4 months 1 day | no |
| Italy | # 711 | S26 | c.221G>A | 2 month 7 days | no |
| Italy | # 803 | S26 | c.3G>A | 8 months 12 days | no |
| Italy | # 839 | L11 | c.60_61delCT | 1 month 6 days | no |
| Italy | # 978 | S26 | c.4-3_4delCAGA | 1 month | unknown |

n.a= not available

**Table S2: Genotyping data of DBA patients. light blue = heterozygous, yellow = homozygous minor allele, -- = no data, white = homozygous major allele**

| **Sources** | **Sample ID** | **rs6198** | **rs6196** | **rs7701443** | **rs6190** | **rs10482616** | **rs860457** | **rs33388** | **rs33389** | **rs10482605** |
| --- | --- | --- | --- | --- | --- | --- | --- | --- | --- | --- |
| Czech | # d1 | AG | AA | AG | GG | GG | TC | AT | CC |  |
| Czech | # d10 | AA | AA | GG | GG | GG | TT | AT | CC | TC |
| Czech | # d11 | AA | AA | AG | GG | GG | TT | AT | CC | TT |
| Czech | # d2 | GG | AA | AA | GG | GG | CC | AA | CC | TT |
| Czech | # d3 | AG | AA | AA | GG | GG | TC | AA | CC | TT |
| Czech | # d4 | AA | AA | AG | GG | GG | TT | AT | CC | TT |
| Czech | # d6 | AA | AG | AA | GG | GG | TC | AA | CT | TT |
| Czech | # d9 | AA | AA | GG | GG | GG | TT | TT | CC | TC |
| France | # 168 | AA | AA | AG | GG | AG | TT |  |  | TT |
| France | # 199 | AA | AG | AA | GG | GG | CC |  |  | TT |
| France | # 215 | AG | AA | AG | GG | GG | TC |  |  |  |
| France | # 227 | AA | AA | GG | GG | GG | TT |  |  | TT |
| France | # 240 | AA | AA | GG | GG | GG | TT |  |  | TT |
| France | # 254 | AA | AG | AG | GG | GG | TC | AT | CT | TT |
| France | # 262 | AA | AG | AG | GG | GG | TC |  |  |  |
| France | # 303 | AA | AG | AG | GG | GG | TC |  |  |  |
| France | # 35 | AA | AG | GG | GG | GG | TC |  |  |  |
| France | # 36 | AA | AA | GG | GG | GG | TT |  |  | TC |
| France | # 367 L | AA | AA | AA | GG | GG | TT | AT | CC | TC |
| France | # 439 | AG | AA | AG |  | GG | TC |  |  |  |
| France | # 50 | AA | AG | AG | GG | GG | TC |  |  |  |
| France | # 501 | AG | AG | AG | GG | GG | CC |  |  | TT |
| France | # 587 | AG | AA | AG | GG | GG | TT |  |  | CC |
| France | # 588 | AG | AA | AA | GG | GG | TC |  |  | TT |
| France | # 590 | AA | AA | AA | GG | GG | TT | AT |  | TT |
| France | # 598 | AA | AA | GG | GG | GG | TT |  |  |  |
| Italy | # 1035 | AA | AA | AG | GG | AG | TT | TT | CC | TC |
| Italy | # 1099 | AA | AA | AG | GG | GG | TT | AT | CC |  |
| Italy | # 1239 | AG | AA | AG | GG | GG | TC | AT | CC | TT |
| Italy | # 1240 | AA | AA | GG | GG | AG | TT | AT | CC | TT |
| Italy | # 1241 | AA | AA | GG | GG | GG | TT | TT | CC | TT |
| Italy | # 1252 | AG | AA | AG | GG | AG | TC | AT | CC | TT |
| Italy | # 1383 | AG | AA | AA |  | GG | TC | AT | CC | TT |
| Italy | # 1389 | AG | AA | AG |  | AG | TC | AT | CC |  |
| Italy | # 1429 | AA | AA | GG | GG | GG | TT | TT | CC | TT |
| Italy | # 1448 | AA | AA | GG | GG | GG | TT | TT | CC |  |
| Italy | # 1459 | AG | AA | AA | GG | GG | TC | AA | CC | TT |
| Italy | # 1460* | AA | AG | AG | GG | GG | TC | AA | CT | TC |
| Italy | # 1466 | AA | AG | AG | GG | GG | TC | AT | CT | TT |
| Italy | # 1491 | AA | AG | AG | GG | GG | TC | AA | CT | TT |
| Italy | # 1504 | AG | AA | AG | GG | GG | TC | AT | CC | TT |
| Italy | # 1506 | AA | AA | AG | GG | AG | TC |  |  | TT |
| Italy | # 1508 | GG | AA | AA | GG | GG | CC | AA | CC | TT |
| Italy | # 1518 | AA | AG | AG |  | GG | TC | AT | CT | TT |
| Italy | # 1529 | AG | AA | AA |  | GG | TC | AA | CC | TT |
| Italy | # 1624 | AG | AA | AG | GG | GG | TC | AT | CC | TT |
| Italy | # 1673 | GG | AA | AA | GG | GG | CC | AA | CC | TT |
| Italy | # 1676 | AA | AA | GG | GG | GG | TT | AT | CC | TT |
| Italy | # 1703 | AA | AA | AA | GG | AG | TT | AT |  | TT |
| Italy | # 1707 | AA | AA | GG | GG | GG | TT | AA | CC | TT |
| Italy | # 1722 |  |  |  |  | GG | TT | AA | CC | TT |
| Italy | # 1816 | AA | AG | AA | GG | GG | TC | AA | CT | TT |
| Italy | # 1830 | AG | AA | AG | GG | GG | TC | AT | CC | TT |
| Italy | # 1904 | AA | AG | AA | GG | GG | TC | AA | CT | TC |
| Italy | # 1929 | AA | AA | GG | GG | GG | TT | AT | CC | TT |
| Italy | # 1945 | AA | AA | GG | GG | AG | TT |  |  | TT |
| Italy | # 1981 | AA | AA | GG | GG | GG | TT | TT | CC | TT |
| Italy | # 2087 |  |  |  |  | GG | TC | AT | CT | TT |
| Italy | # 2124 |  |  |  |  | GG | CC | AT | CC | TT |
| Italy | # 2137 |  |  |  |  | AG | TT | AT | CT | TT |
| Italy | # 2206 |  |  |  |  | GG | TC | AA | CC | TT |
| Italy | # 2221 |  |  |  |  | GG | TT | TT | CC | TC |
| Italy | # 2471 |  |  |  |  | GG | TC | AA | CC | TC |
| Italy | # 272 | AG | AA | AG | GG | GG | TC | AA | CC | TC |
| Italy | # 277 | AG | AG | AA | GG | GG | CC | AA |  |  |
| Italy | # 279 | AA | AG | AA | GG |  | TC |  |  | TC |
| Italy | # 284 | AA |  | AG |  |  |  |  | CT |  |
| Italy | # 293 | AG | AA | AA | GG | AG | TC | AT | CC | TT |
| Italy | # 359 | AG | AA | AG | GG | GG | TC | AT | CC | TT |
| Italy | # 363 | AG |  |  |  |  |  |  |  |  |
| Italy | # 367 R | AA | AG | AG | GG | GG | TC | AT | CT |  |
| Italy | # 383 | AA | AA | AA | GG | GG | TT | AA |  |  |
| Italy | # 387 | AG | AA | AA | GG | GG | TC | AA | CC | TT |
| Italy | # 456* | AG | AA | AA | GG | GG | TC | AA | CC | TT |
| Italy | # 471 | AA | AG | AG |  | GG | TC | AT | CT | TT |
| Italy | # 472 | AA | AG | AG | GG | GG | TC | AT |  | TT |
| Italy | # 476 | AA | AA | AG | GG | GG | TT | AT | CC | TT |
| Italy | # 477 | AA | AA | GG | GG | AG | TT | TT | CC | CC |
| Italy | # 502 | AG | AA | AA | GG | GG | TC | AA | CC | TT |
| Italy | # 518 | AG | AA | GG | GG | GG | TC | AT | CC | TT |
| Italy | # 521 | GG | AA | AA | GG | GG | CC |  |  | TT |
| Italy | # 548* | GG | AA | AA | GG | GG | CC | AA | CC | TC |
| Italy | # 647 | AA | AA | AG | GG | AG |  | AT | CC | TT |
| Italy | # 667 | AA | GG | AA | GG | GG | CC | AA | TT | TC |
| Italy | # 671 | AA | AA | AA | GG | GG | TT | AA | CC | TT |
| Italy | # 672 | AG | AG | AA | GG |  | CC | AA | CT | TC |
| Italy | # 673 | AG | AA | AA | GG | GG | TC | AA | CC | TT |
| Italy | # 711 | AA | AA | AA | GG | GG | TT | AT | CC | TC |
| Italy | # 803 | AA | AG | AG | GG | AG | TC | AT | CT | TT |
| Italy | # 839 | AG | AA | AG | GG | AG | TC | AT | CC | TT |
| Italy | # 978 | AA | AA | AA | GG | AG | TT |  |  | TT |

**Table S3: Genotyping data of Healthy controls. light blue = heterozygous, yellow = homozygous minor allele, -- = no data, white = homozygous major allele**

| **Sources** | **Sample ID** | **rs6198** | **rs6196** | **rs7701443** | **rs6190** | **rs10482616** | **rs860457** | **rs33388** | **rs33389** | **rs10482605** |
| --- | --- | --- | --- | --- | --- | --- | --- | --- | --- | --- |
| USA | # 40 S |  | AG | AA | GG | GG | TC |  |  |  |
| USA | # 41 S |  | AA | GG | GG | GG | TT |  |  |  |
| USA | # 42 S |  | AA | AA | GG | AA | TT |  |  |  |
| USA | # 43 |  | AA | AA | GG | AG | TT |  |  |  |
| USA | # 44 |  | AA | AG |  | AG | TT |  |  |  |
| USA | # 45 |  | AA | AA |  | GG | TT |  |  |  |
| USA | # 46 |  | AG | AG |  | GG | TC |  |  |  |
| USA | # 47 |  | AA | AA |  | AG | TT |  |  |  |
| USA | # 48 |  | AA | AA |  | GG | TC |  |  |  |
| USA | # 49 |  | AA | AG |  | AG | TC |  |  |  |
| Italy | # 21 |  |  |  |  |  |  | AT | CC |  |
| Italy | # 22 |  |  |  |  |  |  | AA | CT |  |
| Italy | # 23 |  |  |  |  |  |  | AA | CC |  |
| Italy | # 25 |  |  |  |  |  |  | AT | CC |  |
| Italy | # 30 |  |  |  |  |  |  | TT | CC |  |
| Italy | # 31 |  |  |  |  |  |  | TT | CC |  |
| Italy | # 34 |  |  |  |  |  |  | TT | CC |  |
| Italy | # 37 |  |  |  |  |  |  | AA | CC |  |
| Italy | # 39 |  |  |  |  |  |  | AA | CC |  |
| Italy | #40 F |  |  |  |  |  |  |  |  | TT |
| Italy | #41 F |  |  |  |  |  |  |  |  | TT |
| Italy | #42 F |  |  |  |  |  |  |  |  | TT |
| France | # 601 | AA | AA | AG | GG | GG | TT | AT | CC |  |
| France | # 438 | AG | AA | AA | GG | GG | TC |  |  |  |
| France | # 253 | AA | AA | AG | GG | AG | TT |  |  |  |
| France | # 686 | AA | AA | GG | GG | AG | TT | AT | CC | TT |
| France | # 232 | AA | AG | AG | GG | GG | TC |  | CC | TT |
| Italy | # 1003 | AA | AA | GG |  | AG | TT | TT | CC | TC |
| Italy | # 1827 | AG | AA | AG |  | GG | TC | AA | CC |  |
| Italy | # 306 | AA |  |  |  |  |  |  |  |  |
| Italy | # 1002 | AA | AA | GG |  | AG | TT | TT | CC | TT |
| Italy | # 1276 | AG | AA | AA | GG | GG | TC | AA | CC |  |
| Italy | # 1277 | AA | AA | GG | GG | GG | TT | AT | CC | TC |
| Italy | # 1532 | AA | AA | GG | GG | GG | TT | TT | CC | TT |
| Italy | # 1591 | AA | AA | AG | GG | GG | TT | TT | CC | TT |
| Italy | # 1619 | AA | AG | AG | GG | GG | TC | AT | CT | TT |
| Italy | # 1994 | AA | AA | AG | GG | GG | TT | AT | CC | TC |

**Table S4: Analysis of SNPs association in DBA vs different control groups (healthly controls, Tuscany population TSI, European population, Italian population NIG) by the chi-squared statistic under several models**

| **Cases *vs* Healthy controls** | | |  |  |  |  |  |  |
| --- | --- | --- | --- | --- | --- | --- | --- | --- |
| **SNP** | **A1** | **A2** | **TEST** | **AFF** | **UNAFF** | **CHISQ** | **DF** | **P** |
| rs6198 | G | A | GENO | 5/28/51 | 0/3/12 | 2.359 | 2 | 0.3074 |
| rs6198 | G | A | TREND | 38/130 | 3/27 | 2.359 | 1 | 0.1246 |
| rs6198 | G | A | ALLELIC | 38/130 | 3/27 | 2.469 | 1 | 0.1161 |
| rs6198 | G | A | DOM | 33/51 | 3/12 | 2.046 | 1 | 0.1526 |
| rs6198 | G | A | REC | 5/79 | 0/15 | 0.9403 | 1 | 0.3322 |
| rs6196 | G | A | GENO | 1/21/60 | 0/4/20 | 1.176 | 2 | 0.5553 |
| rs6196 | G | A | TREND | 23/141 | 4/44 | 1.152 | 1 | 0.283 |
| rs6196 | G | A | ALLELIC | 23/141 | 4/44 | 1.082 | 1 | 0.2982 |
| rs6196 | G | A | DOM | 22/60 | 4/20 | 1.036 | 1 | 0.3088 |
| rs6196 | G | A | REC | 1/81 | 0/24 | 0.2955 | 1 | 0.5867 |
| rs860457 | C | T | GENO | 11/44/33 | 0/9/15 | 6.373 | 2 | **0.04132** |
| rs860457 | C | T | TREND | 66/110 | 9/39 | 6.349 | 1 | **0.01174** |
| rs860457 | C | T | ALLELIC | 66/110 | 9/39 | 5.953 | 1 | **0.01469** |
| rs860457 | C | T | DOM | 55/33 | 9/15 | 4.812 | 1 | **0.02825** |
| rs860457 | C | T | REC | 11/77 | 0/24 | 3.327 | 1 | 0.06816 |
| rs33388 | T | A | GENO | 8/35/26 | 7/7/6 | 6.107 | 2 | **0.0472** |
| rs33388 | T | A | TREND | 51/87 | 21/19 | 3.048 | 1 | 0.08083 |
| rs33388 | T | A | ALLELIC | 51/87 | 21/19 | 3.11 | 1 | 0.07779 |
| rs33388 | T | A | DOM | 43/26 | 14/6 | 0.3973 | 1 | 0.5285 |
| rs33388 | T | A | REC | 8/61 | 7/13 | 6.062 | 1 | **0.01381** |
| rs33389 | T | C | GENO | 1/15/49 | 0/2/19 | 2.255 | 2 | 0.3238 |
| rs33389 | T | C | TREND | 17/113 | 2/40 | 2.247 | 1 | 0.1339 |
| rs33389 | T | C | ALLELIC | 17/113 | 2/40 | 2.234 | 1 | 0.135 |
| rs33389 | T | C | DOM | 16/49 | 2/19 | 2.184 | 1 | 0.1394 |
| rs33389 | T | C | REC | 1/64 | 0/21 | 0.3269 | 1 | 0.5675 |
| rs6190 | 0 | G | GENO | 0/0/76 | 0/0/15 | NA | NA | NA |
| rs6190 | 0 | G | TREND | 0/152 | 0/30 | NA | NA | NA |
| rs6190 | 0 | G | ALLELIC | 0/152 | 0/30 | NA | NA | NA |
| rs6190 | 0 | G | DOM | 0/76 | 0/15 | NA | NA | NA |
| rs6190 | 0 | G | REC | 0/76 | 0/15 | NA | NA | NA |
| rs10482616 | A | G | GENO | 0/15/72 | 1/8/15 | 6.961 | 2 | **0.03079** |
| rs10482616 | A | G | TREND | 15/159 | 10/38 | 5.829 | 1 | **0.01576** |
| rs10482616 | A | G | ALLELIC | 15/159 | 10/38 | 5.615 | 1 | **0.01781** |
| rs10482616 | A | G | DOM | 15/72 | 9/15 | 4.556 | 1 | **0.03281** |
| rs10482616 | A | G | REC | 0/87 | 1/23 | 3.658 | 1 | 0.0558 |
| rs10482605 | C | T | GENO | 2/15/58 | 0/3/9 | 0.4523 | 2 | 0.7976 |
| rs10482605 | C | T | TREND | 19/131 | 3/21 | 0.000489 | 1 | 0.9824 |
| rs10482605 | C | T | ALLELIC | 19/131 | 3/21 | 0.00052 | 1 | 0.9818 |
| rs10482605 | C | T | DOM | 17/58 | 3/9 | 0.03181 | 1 | 0.8584 |
| rs10482605 | C | T | REC | 2/73 | 0/12 | 0.3275 | 1 | 0.5671 |
| rs7701443 | G | A | GENO | 18/35/30 | 6/10/8 | 0.1337 | 2 | 0.9354 |
| rs7701443 | G | A | TREND | 71/95 | 22/26 | 0.1242 | 1 | 0.7245 |
| rs7701443 | G | A | ALLELIC | 71/95 | 22/26 | 0.1421 | 1 | 0.7062 |
| rs7701443 | G | A | DOM | 53/30 | 16/8 | 0.06424 | 1 | 0.7999 |
| rs7701443 | G | A | REC | 18/65 | 6/18 | 0.1175 | 1 | 0.7318 |
|  |  |  |  |  |  |  |  |  |
| **Cases *vs* Tuscany population (TSI)** | | | |  |  |  |  |  |
| **SNP** | **A1** | **A2** | **TEST** | **AFF** | **UNAFF** | **CHISQ** | **DF** | **P** |
| rs6198 | C | T | GENO | 5/28/51 | 7/37/67 | 0.01072 | 2 | 0.9947 |
| rs6198 | C | T | TREND | 38/130 | 51/171 | 0.006455 | 1 | 0.936 |
| rs6198 | C | T | ALLELIC | 38/130 | 51/171 | 0.006801 | 1 | 0.9343 |
| rs6198 | C | T | DOM | 33/51 | 44/67 | 0.002507 | 1 | 0.9601 |
| rs6198 | C | T | REC | 5/79 | 7/104 | 0.01037 | 1 | 0.9189 |
| rs6196 | G | A | GENO | 1/21/60 | 3/23/85 | 1.068 | 2 | 0.5863 |
| rs6196 | G | A | TREND | 23/141 | 29/193 | 0.07316 | 1 | 0.7868 |
| rs6196 | G | A | ALLELIC | 23/141 | 29/193 | 0.07478 | 1 | 0.7845 |
| rs6196 | G | A | DOM | 22/60 | 26/85 | 0.2928 | 1 | 0.5884 |
| rs6196 | G | A | REC | 1/81 | 3/108 | 0.5112 | 1 | 0.4746 |
| rs860457 | C | T | GENO | 11/44/33 | 15/49/47 | 0.6851 | 2 | 0.71 |
| rs860457 | C | T | TREND | 66/110 | 79/143 | 0.1568 | 1 | 0.6922 |
| rs860457 | C | T | ALLELIC | 66/110 | 79/143 | 0.1554 | 1 | 0.6935 |
| rs860457 | C | T | DOM | 55/33 | 64/47 | 0.4788 | 1 | 0.489 |
| rs860457 | C | T | REC | 11/77 | 15/96 | 0.04439 | 1 | 0.8331 |
| rs33388 | T | A | GENO | 8/35/26 | 14/57/40 | 0.07078 | 2 | 0.9652 |
| rs33388 | T | A | TREND | 51/87 | 85/137 | 0.07034 | 1 | 0.7908 |
| rs33388 | T | A | ALLELIC | 51/87 | 85/137 | 0.06421 | 1 | 0.8 |
| rs33388 | T | A | DOM | 43/26 | 71/40 | 0.04959 | 1 | 0.8238 |
| rs33388 | T | A | REC | 8/61 | 14/97 | 0.04113 | 1 | 0.8393 |
| rs33389 | T | C | GENO | 1/15/49 | 3/22/86 | 0.4748 | 2 | 0.7887 |
| rs33389 | T | C | TREND | 17/113 | 28/194 | 0.01499 | 1 | 0.9025 |
| rs33389 | T | C | ALLELIC | 17/113 | 28/194 | 0.01585 | 1 | 0.8998 |
| rs33389 | T | C | DOM | 16/49 | 25/86 | 0.1005 | 1 | 0.7512 |
| rs33389 | T | C | REC | 1/64 | 3/108 | 0.2502 | 1 | 0.617 |
| rs6190 | T | C | GENO | 0/0/76 | 0/4/107 | 2.799 | 1 | 0.09435 |
| rs6190 | T | C | TREND | 0/152 | 4/218 | 2.799 | 1 | 0.09435 |
| rs6190 | T | C | ALLELIC | 0/152 | 4/218 | 2.768 | 1 | 0.09615 |
| rs6190 | T | C | DOM | 0/76 | 4/107 | 2.799 | 1 | 0.09435 |
| rs6190 | T | C | REC | 0/76 | 0/111 | NA | NA | NA |
| rs10482616 | T | C | GENO | 0/15/72 | 1/30/80 | 3.564 | 2 | 0.1683 |
| rs10482616 | T | C | TREND | 15/159 | 32/190 | 3.426 | 1 | 0.06417 |
| rs10482616 | T | C | ALLELIC | 15/159 | 32/190 | 3.13 | 1 | 0.07685 |
| rs10482616 | T | C | DOM | 15/72 | 31/80 | 3.123 | 1 | 0.07719 |
| rs10482616 | T | C | REC | 0/87 | 1/110 | 0.7878 | 1 | 0.3748 |
| rs10482605 | G | A | GENO | 2/15/58 | 7/39/65 | 7.143 | 2 | **0.02812** |
| rs10482605 | G | A | TREND | 19/131 | 53/169 | 6.732 | 1 | **0.00947** |
| rs10482605 | G | A | ALLELIC | 19/131 | 53/169 | 7.203 | 1 | **0.007277** |
| rs10482605 | G | A | DOM | 17/58 | 46/65 | 7.044 | 1 | **0.007954** |
| rs10482605 | G | A | REC | 2/73 | 7/104 | 1.288 | 1 | 0.2565 |
| rs7701443 | G | A | GENO | 18/35/30 | 13/50/48 | 3.642 | 2 | 0.1619 |
| rs7701443 | G | A | TREND | 71/95 | 76/146 | 2.751 | 1 | 0.09717 |
| rs7701443 | G | A | ALLELIC | 71/95 | 76/146 | 2.941 | 1 | 0.08634 |
| rs7701443 | G | A | DOM | 53/30 | 63/48 | 0.9954 | 1 | 0.3184 |
| rs7701443 | G | A | REC | 18/65 | 13/98 | 3.52 | 1 | 0.06065 |
|  |  |  |  |  |  |  |  |  |
| **Cases *vs* European population** | | |  |  |  |  |  |  |
| **SNP** | **A1** | **A2** | **TEST** | **AFF** | **UNAFF** | **CHISQ** | **DF** | **P** |
| rs6198 | C | T | GENO | 5/28/51 | 20/141/361 | 2.585 | 2 | 0.2746 |
| rs6198 | C | T | TREND | 38/130 | 181/863 | 2.577 | 1 | 0.1084 |
| rs6198 | C | T | ALLELIC | 38/130 | 181/863 | 2.727 | 1 | 0.09866 |
| rs6198 | C | T | DOM | 33/51 | 161/361 | 2.37 | 1 | 0.1237 |
| rs6198 | C | T | REC | 5/79 | 20/502 | 0.8229 | 1 | 0.3643 |
| rs6196 | G | A | GENO | 1/21/60 | 16/129/377 | 0.8894 | 2 | 0.641 |
| rs6196 | G | A | TREND | 23/141 | 161/883 | 0.2064 | 1 | 0.6496 |
| rs6196 | G | A | ALLELIC | 23/141 | 161/883 | 0.2143 | 1 | 0.6435 |
| rs6196 | G | A | DOM | 22/60 | 145/377 | 0.03187 | 1 | 0.8583 |
| rs6196 | G | A | REC | 1/81 | 16/506 | 0.8825 | 1 | 0.3475 |
| rs860457 | C | T | GENO | 11/44/33 | 61/218/243 | 2.596 | 2 | 0.2731 |
| rs860457 | C | T | TREND | 66/110 | 340/704 | 1.598 | 1 | 0.2062 |
| rs860457 | C | T | ALLELIC | 66/110 | 340/704 | 1.651 | 1 | 0.1989 |
| rs860457 | C | T | DOM | 55/33 | 279/243 | 2.491 | 1 | 0.1145 |
| rs860457 | C | T | REC | 11/77 | 61/461 | 0.04795 | 1 | 0.8267 |
| rs33388 | T | A | GENO | 8/35/26 | 101/265/156 | 3.19 | 2 | 0.2029 |
| rs33388 | T | A | TREND | 51/87 | 467/577 | 3.089 | 1 | 0.07883 |
| rs33388 | T | A | ALLELIC | 51/87 | 467/577 | 2.993 | 1 | 0.08362 |
| rs33388 | T | A | DOM | 43/26 | 366/156 | 1.738 | 1 | 0.1874 |
| rs33388 | T | A | REC | 8/61 | 101/421 | 2.436 | 1 | 0.1186 |
| rs33389 | T | C | GENO | 1/15/49 | 16/126/380 | 0.5452 | 2 | 0.7614 |
| rs33389 | T | C | TREND | 17/113 | 158/886 | 0.3662 | 1 | 0.5451 |
| rs33389 | T | C | ALLELIC | 17/113 | 158/886 | 0.3857 | 1 | 0.5346 |
| rs33389 | T | C | DOM | 16/49 | 142/380 | 0.1968 | 1 | 0.6574 |
| rs33389 | T | C | REC | 1/64 | 16/506 | 0.4791 | 1 | 0.4888 |
| rs6190 | T | C | GENO | 0/0/76 | 0/30/492 | 4.599 | 1 | **0.032** |
| rs6190 | T | C | TREND | 0/152 | 30/1014 | 4.599 | 1 | **0.032** |
| rs6190 | T | C | ALLELIC | 0/152 | 30/1014 | 4.48 | 1 | **0.03429** |
| rs6190 | T | C | DOM | 0/76 | 30/492 | 4.599 | 1 | **0.032** |
| rs6190 | T | C | REC | 0/76 | 0/522 | NA | NA | NA |
| rs10482616 | T | C | GENO | 0/15/72 | 8/119/395 | 2.868 | 2 | 0.2384 |
| rs10482616 | T | C | TREND | 15/159 | 135/909 | 2.615 | 1 | 0.1058 |
| rs10482616 | T | C | ALLELIC | 15/159 | 135/909 | 2.566 | 1 | 0.1092 |
| rs10482616 | T | C | DOM | 15/72 | 127/395 | 2.095 | 1 | 0.1477 |
| rs10482616 | T | C | REC | 0/87 | 8/514 | 1.351 | 1 | 0.2451 |
| rs10482605 | G | A | GENO | 2/15/58 | 21/142/359 | 2.295 | 2 | 0.3175 |
| rs10482605 | G | A | TREND | 19/131 | 184/860 | 2.139 | 1 | 0.1436 |
| rs10482605 | G | A | ALLELIC | 19/131 | 184/860 | 2.285 | 1 | 0.1307 |
| rs10482605 | G | A | DOM | 17/58 | 163/359 | 2.281 | 1 | 0.1309 |
| rs10482605 | G | A | REC | 2/73 | 21/501 | 0.3257 | 1 | 0.5682 |
| rs7701443 | G | A | GENO | 18/35/30 | 77/249/196 | 2.699 | 2 | 0.2594 |
| rs7701443 | G | A | TREND | 71/95 | 403/641 | 1.03 | 1 | 0.3103 |
| rs7701443 | G | A | ALLELIC | 71/95 | 403/641 | 1.045 | 1 | 0.3067 |
| rs7701443 | G | A | DOM | 53/30 | 326/196 | 0.06027 | 1 | 0.8061 |
| rs7701443 | G | A | REC | 18/65 | 77/445 | 2.603 | 1 | 0.1067 |
|  |  |  |  |  |  |  |  |  |
| **Cases *vs* Italian population (NIG)** | | | |  |  |  |  |  |
| **SNP** | **A1** | **A2** | **TEST** | **AFF** | **UNAFF** | **CHISQ** | **DF** | **P** |
| rs6196 | G | A | GENO | 1/21/60 | 19/259/1404 | 6.142 | 2 | **0.04638** |
| rs6196 | G | A | TREND | 23/141 | 297/3067 | 4.932 | 1 | **0.02636** |
| rs6196 | G | A | ALLELIC | 23/141 | 297/3067 | 5.118 | 1 | **0.02368** |
| rs6196 | G | A | DOM | 22/60 | 278/1404 | 5.878 | 1 | **0.01533** |
| rs6196 | G | A | REC | 1/81 | 19/1663 | 0.005638 | 1 | 0.9401 |
| rs6190 | T | C | GENO | 0/0/76 | 1/64/1621 | 3.042 | 2 | 0.2185 |
| rs6190 | T | C | TREND | 0/152 | 66/3306 | 2.997 | 1 | 0.08344 |
| rs6190 | T | C | ALLELIC | 0/152 | 66/3306 | 3.032 | 1 | 0.08164 |
| rs6190 | T | C | DOM | 0/76 | 65/1621 | 3.042 | 1 | 0.08112 |
| rs6190 | T | C | REC | 0/76 | 1/1685 | 0.0451 | 1 | 0.8318 |
|  |  |  |  |  |  |  |  |  |
| **Responders vs Non-Responders** | | | |  |  |  |  |  |
| **SNP** | **A1** | **A2** | **TEST** | **AFF** | **UNAFF** | **CHISQ** | **DF** | **P** |
| rs6198 | G | A | GENO | 2/12/17 | 2/16/25 | 0.1533 | 2 | 0.9262 |
| rs6198 | G | A | TREND | 16/46 | 20/66 | 0.131 | 1 | 0.7174 |
| rs6198 | G | A | ALLELIC | 16/46 | 20/66 | 0.1273 | 1 | 0.7212 |
| rs6198 | G | A | DOM | 14/17 | 18/25 | 0.07997 | 1 | 0.7773 |
| rs6198 | G | A | REC | 2/29 | 2/41 | 0.1142 | 1 | 0.7354 |
| rs6196 | G | A | GENO | 0/6/24 | 1/11/30 | 1.17 | 2 | 0.5572 |
| rs6196 | G | A | TREND | 6/54 | 13/71 | 0.9455 | 1 | 0.3309 |
| rs6196 | G | A | ALLELIC | 6/54 | 13/71 | 0.9164 | 1 | 0.3384 |
| rs6196 | G | A | DOM | 6/24 | 12/30 | 0.6857 | 1 | 0.4076 |
| rs6196 | G | A | REC | 0/30 | 1/41 | 0.7243 | 1 | 0.3947 |
| rs860457 | C | T | GENO | 4/19/11 | 6/21/17 | 0.5121 | 2 | 0.7741 |
| rs860457 | C | T | TREND | 27/41 | 33/55 | 0.08603 | 1 | 0.7693 |
| rs860457 | C | T | ALLELIC | 27/41 | 33/55 | 0.07886 | 1 | 0.7788 |
| rs860457 | C | T | DOM | 23/11 | 27/17 | 0.3291 | 1 | 0.5662 |
| rs860457 | C | T | REC | 4/30 | 6/38 | 0.06011 | 1 | 0.8063 |
| rs33388 | T | A | GENO | 3/13/12 | 4/18/13 | 0.2142 | 2 | 0.8984 |
| rs33388 | T | A | TREND | 19/37 | 26/44 | 0.1508 | 1 | 0.6978 |
| rs33388 | T | A | ALLELIC | 19/37 | 26/44 | 0.14 | 1 | 0.7083 |
| rs33388 | T | A | DOM | 16/12 | 22/13 | 0.2122 | 1 | 0.645 |
| rs33388 | T | A | REC | 3/25 | 4/31 | 0.008036 | 1 | 0.9286 |
| rs33389 | T | C | GENO | 0/5/22 | 1/7/24 | 1.004 | 2 | 0.6054 |
| rs33389 | T | C | TREND | 5/49 | 9/55 | 0.6289 | 1 | 0.4278 |
| rs33389 | T | C | ALLELIC | 5/49 | 9/55 | 0.6462 | 1 | 0.4215 |
| rs33389 | T | C | DOM | 5/22 | 8/24 | 0.3581 | 1 | 0.5496 |
| rs33389 | T | C | REC | 0/27 | 1/31 | 0.8583 | 1 | 0.3542 |
| rs6190 | 0 | G | GENO | 0/0/27 | 0/0/39 | NA | NA | NA |
| rs6190 | 0 | G | TREND | 0/54 | 0/78 | NA | NA | NA |
| rs6190 | 0 | G | ALLELIC | 0/54 | 0/78 | NA | NA | NA |
| rs6190 | 0 | G | DOM | 0/27 | 0/39 | NA | NA | NA |
| rs6190 | 0 | G | REC | 0/27 | 0/39 | NA | NA | NA |
| rs10482616 | A | G | GENO | 0/8/27 | 0/5/38 | 1.752 | 1 | 0.1857 |
| rs10482616 | A | G | TREND | 8/62 | 5/81 | 1.752 | 1 | 0.1857 |
| rs10482616 | A | G | ALLELIC | 8/62 | 5/81 | 1.593 | 1 | 0.207 |
| rs10482616 | A | G | DOM | 8/27 | 5/38 | 1.752 | 1 | 0.1857 |
| rs10482616 | A | G | REC | 0/35 | 0/43 | NA | NA | NA |
| rs10482605 | C | T | GENO | 1/6/24 | 1/6/28 | 0.06551 | 2 | 0.9678 |
| rs10482605 | C | T | TREND | 8/54 | 8/62 | 0.05854 | 1 | 0.8088 |
| rs10482605 | C | T | ALLELIC | 8/54 | 8/62 | 0.06712 | 1 | 0.7956 |
| rs10482605 | C | T | DOM | 7/24 | 7/28 | 0.06551 | 1 | 0.798 |
| rs10482605 | C | T | REC | 1/30 | 1/34 | 0.007604 | 1 | 0.9305 |
| rs7701443 | G | A | GENO | 7/11/12 | 8/20/15 | 0.7207 | 2 | 0.6974 |
| rs7701443 | G | A | TREND | 25/35 | 36/50 | 0.000484 | 1 | 0.9824 |
| rs7701443 | G | A | ALLELIC | 25/35 | 36/50 | 0.000546 | 1 | 0.9814 |
| rs7701443 | G | A | DOM | 18/12 | 28/15 | 0.1985 | 1 | 0.656 |
| rs7701443 | G | A | REC | 7/23 | 8/35 | 0.242 | 1 | 0.6227 |
|  |  |  |  |  |  |  |  |  |
| **Diagnosis at 4 months** | | |  |  |  |  |  |  |
| **SNP** | **A1** | **A2** | **TEST** | **AFF** | **UNAFF** | **CHISQ** | **DF** | **P** |
| rs6198 | G | A | GENO | 4/19/29 | 1/8/18 | 1.05 | 2 | 0.5917 |
| rs6198 | G | A | TREND | 27/77 | 10/44 | 1.049 | 1 | 0.3058 |
| rs6198 | G | A | ALLELIC | 27/77 | 10/44 | 1.098 | 1 | 0.2947 |
| rs6198 | G | A | DOM | 23/29 | 9/18 | 0.8758 | 1 | 0.3494 |
| rs6198 | G | A | REC | 4/48 | 1/26 | 0.4769 | 1 | 0.4898 |
| rs6196 | G | A | GENO | 1/16/34 | 0/3/23 | 4.36 | 2 | 0.113 |
| rs6196 | G | A | TREND | 18/84 | 3/49 | 4.332 | 1 | **0.0374** |
| rs6196 | G | A | ALLELIC | 18/84 | 3/49 | 4.126 | 1 | **0.04223** |
| rs6196 | G | A | DOM | 17/34 | 3/23 | 4.254 | 1 | **0.03915** |
| rs6196 | G | A | REC | 1/50 | 0/26 | 0.5165 | 1 | 0.4723 |
| rs860457 | C | T | GENO | 9/27/15 | 1/11/13 | 4.966 | 2 | 0.08348 |
| rs860457 | C | T | TREND | 45/57 | 13/37 | 4.962 | 1 | **0.02591** |
| rs860457 | C | T | ALLELIC | 45/57 | 13/37 | 4.667 | 1 | **0.03074** |
| rs860457 | C | T | DOM | 36/15 | 12/13 | 3.679 | 1 | 0.05511 |
| rs860457 | C | T | REC | 9/42 | 1/24 | 2.734 | 1 | 0.09821 |
| rs33388 | T | A | GENO | 3/21/16 | 3/11/6 | 1.129 | 2 | 0.5686 |
| rs33388 | T | A | TREND | 27/53 | 17/23 | 1.032 | 1 | 0.3096 |
| rs33388 | T | A | ALLELIC | 27/53 | 17/23 | 0.8792 | 1 | 0.3484 |
| rs33388 | T | A | DOM | 24/16 | 14/6 | 0.5742 | 1 | 0.4486 |
| rs33388 | T | A | REC | 3/37 | 3/17 | 0.8333 | 1 | 0.3613 |
| rs33389 | T | C | GENO | 1/10/29 | 0/2/14 | 1.568 | 2 | 0.4565 |
| rs33389 | T | C | TREND | 12/68 | 2/30 | 1.568 | 1 | 0.2105 |
| rs33389 | T | C | ALLELIC | 12/68 | 2/30 | 1.6 | 1 | 0.2059 |
| rs33389 | T | C | DOM | 11/29 | 2/14 | 1.443 | 1 | 0.2297 |
| rs33389 | T | C | REC | 1/39 | 0/16 | 0.4073 | 1 | 0.5234 |
| rs6190 | 0 | G | GENO | 0/0/46 | 0/0/25 | NA | NA | NA |
| rs6190 | 0 | G | TREND | 0/92 | 0/50 | NA | NA | NA |
| rs6190 | 0 | G | ALLELIC | 0/92 | 0/50 | NA | NA | NA |
| rs6190 | 0 | G | DOM | 0/46 | 0/25 | NA | NA | NA |
| rs6190 | 0 | G | REC | 0/46 | 0/25 | NA | NA | NA |
| rs10482616 | A | G | GENO | 0/6/44 | 0/7/19 | 2.687 | 1 | 0.1012 |
| rs10482616 | A | G | TREND | 6/94 | 7/45 | 2.687 | 1 | 0.1012 |
| rs10482616 | A | G | ALLELIC | 6/94 | 7/45 | 2.435 | 1 | 0.1186 |
| rs10482616 | A | G | DOM | 6/44 | 7/19 | 2.687 | 1 | 0.1012 |
| rs10482616 | A | G | REC | 0/50 | 0/26 | NA | NA | NA |
| rs10482605 | C | T | GENO | 2/7/31 | 0/4/20 | 1.27 | 2 | 0.5299 |
| rs10482605 | C | T | TREND | 11/69 | 4/44 | 0.7276 | 1 | 0.3937 |
| rs10482605 | C | T | ALLELIC | 11/69 | 4/44 | 0.8508 | 1 | 0.3563 |
| rs10482605 | C | T | DOM | 9/31 | 4/20 | 0.3153 | 1 | 0.5744 |
| rs10482605 | C | T | REC | 2/38 | 0/24 | 1.239 | 1 | 0.2657 |
| rs7701443 | G | A | GENO | 9/23/20 | 8/9/9 | 1.901 | 2 | 0.3866 |
| rs7701443 | G | A | TREND | 41/63 | 25/27 | 0.9172 | 1 | 0.3382 |
| rs7701443 | G | A | ALLELIC | 41/63 | 25/27 | 1.064 | 1 | 0.3024 |
| rs7701443 | G | A | DOM | 32/20 | 17/9 | 0.1098 | 1 | 0.7404 |
| rs7701443 | G | A | REC | 9/43 | 8/18 | 1.843 | 1 | 0.1746 |

**Table S5: List of endpoints included in the RPPA array and of their fold changes in erythroid cells expanded with and without Dex either from adult blood.** Hits that met the threshold for upregulation (Fold Change <0.5) and downregulation (Fold Change >2) are in red and in green fonts, respectively. Statistically significant p values (p < 0.05 by Wilcoxon test) are shown in bold. (For further details see: http://capmm.gmu.edu/data) . Concordant and non-concordant effects of Dex with respect to those shown for cord blood in Table S6 are indicates by → and ← respectively.

|  | | **AB day10** | |
| --- | --- | --- | --- |
| **Gene ID** | **Protein** | **Fold Change AB day10 - Dex/AB day10 + Dex** | **Prob>ChiSq** |
| EIF4EBP1 | 4E-BP1 (S65) | 0.39 | 0.2752 |
| ACACA | Acetyl-CoA Carboxylase (S79) | 0.39 | 0.1266 |
| AKT1 | AKT (T308) | 0.42 | 0.2683 |
| ALDH1 | ALDH | 2.28 | 0.8273 |
| **ALK** | ALK | 0.32 | **0.0495** |
| **ALK** | ALK (Y1586) | 0.09 | **0.0463** |
| ALK | ALK (Y1604) | 0.28 | 0.1266 |
| **PRKAB1** | AMPK beta1 (S108) | 0.50 | **0.0495** |
| AR | Androgen Receptor (S81) | 0.49 | 0.1266 |
| MAP3K5 | ASK1 (S83) | 0.43 | 0.2752 |
| ATF2 | ATF-2 (T71) | 0.48 | 0.5127 |
| **AURKA/B/C** | Aurora A (T288)/B (T232)/C (T198) | 0.60 | **0.0495** |
| **BAX** | Bax | 1.50 | **0.0495** |
| **BCL2** | Bcl-2 (T56) | 0.81 | **0.0495** |
| **KIT** | c-Kit | 0.23 | **0.0495** |
| **KIT** | c-Kit (Y703) | 0.12 | **0.0495** |
| **KIT** | c-Kit (Y719) | 0.28 | **0.0495** |
| CASP3 | cleaved Caspase 3 (D175) | 5.54 | 0.2752 |
| CASP6 | cleaved Caspase 6 (D162) | 2.29 | 0.8273 |
| **CASP7** | cleaved Caspase 7 (D198) | 32.19 | **0.0495** |
| **SPTAN1** | cleaved Fodrin alpha (D1185) | 2.39 | **0.0495** |
| PARP1 | cleaved PARP (D214) | 2.32 | 0.1266 |
| **CFL1** | Cofilin (S3) | 5.02 | **0.0495** |
| **CCNA** | Cyclin A | 1.47 | **0.0495** |
| **CCNB1** | Cyclin B1 | 0.54 | **0.0495** |
| **CYCS** | Cytochrome C | 1.89 | **0.0495** |
| **DEPTOR** | DEPTOR | 0.66 | **0.0495** |
| CDH1 | E-Cadherin | 0.40 | 0.1266 |
| **EGFR** | EGFR | 0.77 | **0.0495** |
| **EGFR** | EGFR (Y1068) | 0.43 | **0.0495** |
| **HER2** | ErbB2 | 1.73 | **0.0495** |
| **HER2** | ErbB2 (Y1248) | 1.14 | **0.0495** |
| **FADD** | FADD (S194) | 0.29 | **0.0495** |
| **PTK2** | FAK (Y576/577) | 0.60 | **0.0495** |
| **FOXO1/O3** | FKHR (T24)/FKHRL1 (T32) | 2.87 | **0.0495** |
| **FRS2** | FRS2 alpha (Y436) | 0.38 | **0.0495** |
| FYN | Fyn | 2.08 | 0.2752 |
| **NR3C1** | Glucocorticoid Receptor (S211) | 1.66 | **0.0495** |
| **GSK3A** | GSK-3 alpha/beta (S279/216) | 0.50 | **0.0495** |
| **IGF1R/INSR** | IGF-1R (Y1131)/IR (Y1146) | 1.46 | **0.0495** |
| **NFKBIA** | IkB alpha (S32/36) | 1.26 | **0.0463** |
| **MKI67** | KI67 | 0.73 | **0.0495** |
| **LCK** | Lck (Y505) | 3.27 | **0.0495** |
| STK11 | LKB1 (S428) | 0.50 | 0.2752 |
| **MARCKS** | MARCKS (S152/156) | 2.73 | **0.0495** |
| **RPS6KA5** | MSK1 (S360) | 1.90 | **0.0495** |
| **NOTCH1** | Notch1 | 1.17 | **0.0495** |
| **NPM1** | NPM (T199) | 0.70 | **0.0495** |
| **SPP1** | Osteopontin | 0.74 | **0.0495** |
| MAPK14 | p38 MAPK (T180/Y182) | 0.06 | 0.1266 |
| **PDGFRB** | PDGFR beta (Y716) | 0.20 | **0.0495** |
| **PDGFRB** | PDGFR beta (Y751) | 0.63 | **0.0495** |
| **PRKCD** | PKC delta (T505) | 1.39 | **0.0495** |
| **PLCG1** | PLCgamma1 (Y783) | 0.53 | **0.0495** |
| **AKT1S1** | PRAS40 (T246) | 1.35 | **0.0495** |
| **RET** | Ret (Y905) | 0.15 | **0.0495** |
| **RPS6KA2** | RSK3 (T356/S360) | 2.18 | **0.0495** |
| **MAPK11/MAPK8** | SAPK/JNK (T183/Y185) | 0.43 | **0.0495** |
| **SHC1** | Shc (Y317) | 0.40 | **0.0495** |
| **DIABLO** | Smac/Diablo | 0.76 | **0.0495** |
| **SMAD2** | Smad2 (S245/250/255) | 0.23 | **0.0495** |
| **STAT1** | STAT1 (Y701) | 12.86 | **0.0463** |
| **STAT3** | STAT3 (Y705) | 6.89 | **0.0495** |
| **BIRC5** | Survivin | 0.68 | **0.0495** |
| **TSC2** | Tuberin/TSC2 (Y1571) | 0.81 | **0.0495** |
| **VAV3** | Vav3 (Y173) | 0.57 | **0.0495** |
| **KDR** | VEGFR2 (Y996) | 0.56 | **0.0495** |

**Table S6: List of endpoints included in the RPPA array and of their fold changes in erythroid cells expanded with and without Dex either from cord blood.** Hits that met the threshold for upregulation (Fold Change <0.5) and downregulation (Fold Change >2) are in red and in green fonts, respectively. Statistically significant p values (p < 0.05 by Wilcoxon test) are shown in bold. (For further details see: http://capmm.gmu.edu/data) . Concordant and non-concordant effects of Dex with respect to those shown for adult blood in Table S5 are indicates by → and ← respectively.

|  | | **CB day10** | |
| --- | --- | --- | --- |
| **Gene ID** | **Protein** | **Fold Change CB day10 - Dex/CB day10 + Dex** | **Prob>ChiSq** |
| **EIF4EBP1** | 4E-BP1 (T37/46) | 1.42 | **0.0495** |
| **EIF4EBP1** | 4E-BP1 (T70) | 1.89 | **0.0495** |
| **ARAF** | A-Raf (S299) | 1.47 | **0.0495** |
| ATF2 | ATF-2 (T69/71) | 2.49 | 0.1266 |
| **BMI1** | Bmi-1 | 2.65 | **0.0495** |
| **ABL** | c-Abl (Y245) | 2.88 | **0.0495** |
| CASP3 | cleaved Caspase 3 (D175) | 0.21 | 0.2752 |
| **CFL1** | Cofilin (S3) | 0.17 | **0.0495** |
| **PTGS2** | Cox2 | 0.50 | **0.0495** |
| **CRKL** | CrkL (Y207) | 0.45 | **0.0495** |
| EIFG41 | eIF4G (S1108) | 0.24 | 0.5127 |
| **HER2** | ErbB2 | 0.57 | **0.0495** |
| **FOXO1/O3** | FKHR (T24)/FKHRL1 (T32) | 0.41 | **0.0495** |
| **NR3C1** | Glucocorticoid Receptor (S211) | 0.72 | **0.0495** |
| **JAK1** | Jak1 (Y1022/1023) | 0.59 | **0.0495** |
| **STK11** | LKB1 (S334) | 1.34 | **0.0495** |
| STK11 | LKB1 (S428) | 0.39 | 0.5127 |
| **RPS6KA5** | MSK1 (S360) | 1.30 | **0.0495** |
| **MTOR** | mTOR (S2448) | 0.57 | **0.0495** |
| **NOTCH1** | Notch1 | 1.30 | **0.0495** |
| MAPK14 | p38 MAPK (T180/Y182) | 2.59 | 0.2752 |
| **AKT1S1** | PRAS40 (T246) | 1.37 | **0.0495** |
| **PTEN** | PTEN | 1.24 | **0.0495** |
| **PTEN** | PTEN (S380) | 1.22 | **0.0495** |
| **TNFSF11** | RANKL | 0.62 | **0.0495** |
| RPS6KA1 | S6 Ribosomal Protein (S235/236) | 0.25 | 0.2752 |
| **STAT3** | STAT3 (Y705) | 2.05 | **0.0495** |
| **VIM** | Vimentin | 1.45 | **0.0495** |
| XIAP | XIAP | 4.97 | 0.1212 |
